# Unlocking the Therapeutic Applicability of LNP-mRNA: Chemistry, Formulation, and Clinical Strategies

**DOI:** 10.34133/research.0370

**Published:** 2024-06-18

**Authors:** Xiaonan Huang, Yishan Ma, Guanghui Ma, Yufei Xia

**Affiliations:** ^1^ Sinovac Biotech Ltd., Beijing, PR China.; ^2^ State Key Laboratory of Biochemical Engineering, Institute of Process Engineering, Chinese Academy of Sciences, Beijing, PR China.; ^3^ School of Chemical Engineering, University of Chinese Academy of Sciences, Beijing, PR China.

## Abstract

Messenger RNA (mRNA) has emerged as an innovative therapeutic modality, offering promising avenues for the prevention and treatment of a variety of diseases. The tremendous success of mRNA vaccines in effectively combatting coronavirus disease 2019 (COVID-19) evidences the unlimited medical and therapeutic potential of mRNA technology. Overcoming challenges related to mRNA stability, immunogenicity, and precision targeting has been made possible by recent advancements in lipid nanoparticles (LNPs). This review summarizes state-of-the-art LNP-mRNA-based therapeutics, including their structure, material compositions, design guidelines, and screening principles. Additionally, we highlight current preclinical and clinical trends in LNP-mRNA therapeutics in a broad range of treatments in ophthalmological conditions, cancer immunotherapy, gene editing, and rare-disease medicine. Particular attention is given to the translation and evolution of LNP-mRNA vaccines into a broader spectrum of therapeutics. We explore concerns in the aspects of inadequate extrahepatic targeting efficacy, elevated doses, safety concerns, and challenges of large-scale production procedures. This discussion may offer insights and perspectives on near- and long-term clinical development prospects for LNP-mRNA therapeutics.

## Introduction

The development of messenger RNA (mRNA) vaccines by Pfizer-BioNTech and Moderna against the 2019 coronavirus disease (COVID-19) represented substantial strides in the evolution of mRNA therapy [1,2]. Over the last century, nucleic acid has shown great potential over traditional protein-based therapeutics. Among all types of nucleic acid, mRNA medicine refers to the introduction of genetic information in the form of mRNA for protein production. which could be used for direct or indirect therapeutic purposes. Initially, limited trials explored mRNA’s functionality and clinical efficacy. In the 1990s, the first published paper reported protein production after the injection of in vitro-transcribed (IVT) mRNA into mice [3], followed by various studies in the 1990s to 2000s [4]. However, due to mRNA’s unstable nature, high innate immunogenicity, and inadequate in vivo delivery efficacy, it garnered less attention as a therapeutic alternative back then. Along the journey, substantial effort has driven mRNA modality into drug candidates, revolutionizing medical interventions for both large populations and individuals. Optimizing nucleotide sequence, modifying nucleotide structure, and installing cap motif have consecutively contributed to the enhanced on-shelf and in vivo stability of mRNA drug molecules. While innate immunogenicity benefits only mRNA vaccines, it can be detrimental when mRNA medicines function as therapeutics for protein replacement. The substitution of pseudo-uridine has resolved this, moderating the immune responses [5]. In addition, major technological advances have expanded mRNA medicines beyond vaccines, making it a promising prophylactic and therapeutic modality empowered by progress and breakthroughs in molecular biology, materials engineering, and chemical engineering.

In the early 21st century, research investors began to pay attention to overcoming the hurdles in utilizing synthetic mRNA to fight diseases and regulate cell functions, by prompting protein production in vivo. Long before the 2019 pandemic, the concept of harnessing mRNA for solving complex healthcare problems had already captured scientific interest [6,7]. Specifically, the 2023 Nobel Prize in Physiology or Medicine recognized Katalin Karikó and Drew Weissman for their contribution in modifying mRNA, enabling a platform for rapidly developing life-saving vaccines during the global COVID-19 crisis. As an effective medicine, mRNA is expected to effectively treat various diseases, especially refractory conditions. In theory, mRNA therapeutics hold the potential to prevent, diagnose, or treat any disease that is involved with genetic components within the cell. While it may initially appear confined to genetic regulation, mRNA may comprehensively address disorders related to in vivo protein production. Under these circumstances, a wide range of diseases, including vaccines, metabolic diseases, cardiovascular and cerebrovascular diseases, as well as cancer, is expected to benefit from mRNA-based therapeutics [8–10]. Despite the limitation of transient protein expression, they may possess advantages in safety, efficacy, and easier manufacture, compared to traditional protein and DNA drugs [11–14]. The recent surge in more investor interest and billions of research funding underscores the therapeutic applicability of mRNA medicine as one of the most appealing scopes for development.

While the conceptual steps in mRNA therapy development may seem rapid and straightforward, the establishment of highly effective mRNA medicines requires more than routine processes. This includes tasks, such as locating the desired protein with its amino acid sequence, designing the mRNA sequence, manufacturing encapsulated mRNA with a suitable delivery system, and ensuring quality control during large-scale production. Each step necessitates delicate design with interdisciplinary techniques. In that, numerous articles intensively reviewed and updated the advances in tailoring the nucleotide sequence and backbone structure of mRNA, as well as their immense potential for enhanced stability, protein production, and therapeutic efficacy [8–10,15–17]. Here, we primarily focus on the delivery vehicle to broaden the applicability of mRNA medicines for common and rare diseases. Aiming to offer design guidelines from a clinical perspective, we put more emphasis on translational strategies to increase mRNA stability during production and administration and, more importantly, facilitate mRNA transport into the targeted organs beyond muscle and liver.

Over the past decades, various drug delivery systems, such as biodegradable nanoparticles, liposomes, and hydrogels, have been applied for mRNA medicines [18–21]. Among these, lipid nanoparticles (LNPs) serve as the most widely used delivery systems for mRNA, with over a billion doses of LNP-mRNA vaccines administered globally. LNPs have been proven to be safe and highly efficacious, with rapid, cost-effective, and scalable manufacturing and along with transient inherent immunogenicity. In addition, LNP-mRNA has emerged as therapeutics and has been leveraged in multiple clinical trials, involving applications such as cancer immunotherapy, protein replacement therapy, and gene editing. Several review articles generally and systemically discuss the advances of LNP technologies, offering insightful perspectives for LNP applications in mRNA delivery [7,10,22].

However, scant attention and critical values have been devoted to discerning the precise mechanisms through which LNPs catalyze the advancement of mRNA medicines or address the remaining unmet need in realizing “undruggable” mRNA therapeutics. Furthermore, a distinct niche pertains to the translation and evolution of LNP-mRNA vaccines into a broader spectrum of LNP-mRNA therapeutics. A series of challenges must be tackled to establish LNP-mRNA as a general therapeutic approach with broad applicability to both rare and common diseases. (a) Dose issue and chemistry, manufacturing, and controls (CMC) requirement: While minimal protein expression suffices as an antigenic signal for the immune system in mRNA vaccines, conventional doses of mRNA-based drugs, such as protein replacement therapy and gene-editing therapeutics, require over 1000-fold or higher amounts, resulting in substantially increased costs and harsher CMC requirements [23]. (b) Safety concern: Higher dosages bring out increased pressure on absorption, distribution, metabolism, and excretion (ADME), raising safety concerns regarding the synthetic lipids within the LNP-mRNA formulations, especially novel ionizable lipid and LNPs formula. (c) Unmet needs in targeting efficiency: Unlike locally administered vaccines, mRNA drugs demand enhanced cargo delivery to target organs and cells, particularly for gene-altering and epigenetic-regulated therapeutics. Anatomical and biological barriers, as well as serum degradation and clearance, pose formidable obstacles to the precise targeting of LNP-mRNA. Although various new delivery systems have been developed to achieve a longer circulatory half-life and carry the cargo to the tissue of interest, the liver still occupies the most readily targeting tissue via intravenous delivery. Ongoing efforts in extrahepatic carriers never stop [24,25], yet limited bioavailability of solid organ and invasive administrative routes restrict the efficacy, duration, and clinical potential.

**Fig. 1.**
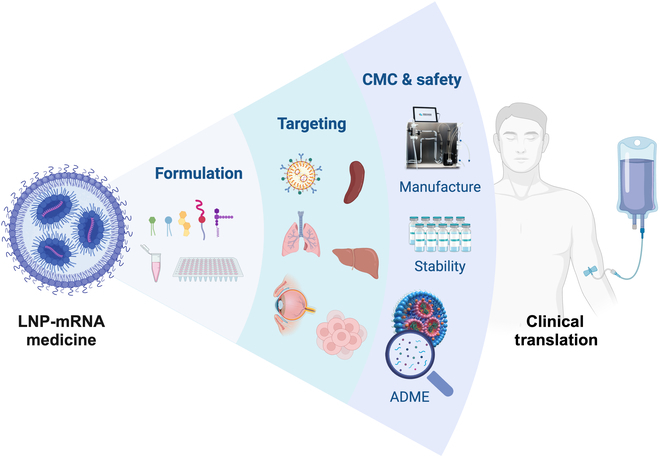
Establishing chemistry, formulation, targeted delivery, and CMC approaches to tackle the hurdles hindering LNP-mRNA clinical translation. Created with BioRender.com.

Here, we reviewed and discussed the state of the art of LNP-mRNA, examining potential strategies to overcome obstacles hindering the clinical translation of mRNA medicines. Our exploration covered key aspects, including LNP formulations, targeting strategies, as well as the large-scale production and characterization procedures (Fig. 1). We initiated with an overview of recent advancements in ionizable lipid, cholesterol, phosphate lipid, and PEGylated lipid, elucidating their compositions and showcasing the structural impact on LNP formulations for the enhanced mRNA encapsulation, stability, and delivery. Additionally, besides the formulation, we addressed the influence of protein adsorption and ligand–receptor interactions on targeting efficiency. Our discussion also encompassed the emerging techniques and novel packing systems to realize specific tissue targeting and tropism. Next, we presented insights into the manufacturing and storage process, exploring long-term and room-temperature storage of LNP-mRNA formulations. Novel strategies for single-particle level LNP analysis, as well as ADME considerations from an industrial perspective, were also demonstrated. Finally, we provided a comprehensive summary of current preclinical and clinical trends in mRNA therapeutics, covering lipid metabolism, safety issues, mode of action for LNPs, as well as the prospects for LNP-mRNA treatment in ophthalmological conditions, cancer immunotherapies, gene editing-based therapies, and rare-disease conditions. This review aims to offer perspectives, guiding principles, and scopes for near- and long-term clinical development of innovative mRNA therapeutic modalities, shedding light on in-depth research and clinical applications of novel LNPs for safe and efficient treatment against chronic and rare diseases.

## Lipid and LNP Formulations for mRNA Medicines

In past decades, intensive research outcomes have nurtured modern LNP technology, currently standing as the most advanced nonviral nucleic acid delivery system in clinical applications, exemplified by US Food and Drug Administration (FDA)-approved LNP-RNA therapies such as patisiran. Patisiran (Onpattro), administered intravenously, facilitates the delivery of a 0.3 mg/kg dose of small interfering RNA (siRNA) to hepatocytes [26]. The approval and scaling-up for Onpattro have paved a way for the rapid subsequent development and production of LNP-mRNA vaccines. Notably, these vaccines have earned distinction as the most expeditiously produced vaccines in history and constitute a pivotal component in the global endeavor to combat COVID-19. Besides the three approved LNP-RNA medicines and vaccines, countable clinical trials are presently investigating the utilization of LNP for mRNA therapeutics in various conditions, including the first in vivo application of CRISPR/Cas9 treatment administered intravenously for the management of patients with transthyretin amyloidosis (ATTR amyloidosis) [27]. Consequently, LNPs emerge as a versatile platform for mRNA delivery at surmounting pivotal challenges in the medical applications such as gene therapy and protein replacement, specifically addressing concerns related to mRNA degradation and constrained cellular uptake in the organ of interest. However, despite the advancements and advantages, there are no distinct guidelines for LNP creation, optimization, and production for effectively loading and delivering respective mRNA to treat specific condition. In particular, it is noteworthy that no singular LNP formulation universally caters to all applications. The discovery and development of ionizable lipids benefit the efficient mRNA encapsulation and finalization, while the optimization of the LNP formulations and surface modifications largely promotes the specific and targeted delivery to the organ of interest for mRNA medicines to treat specific diseases beyond liver and vaccines. Therefore, in this section, we summarized the bumping journey of LNP development in the aspect of lipid chemistry and LNP formulations, illustrating the most advanced progress for various mRNA therapeutic applications.

**Fig. 2.**
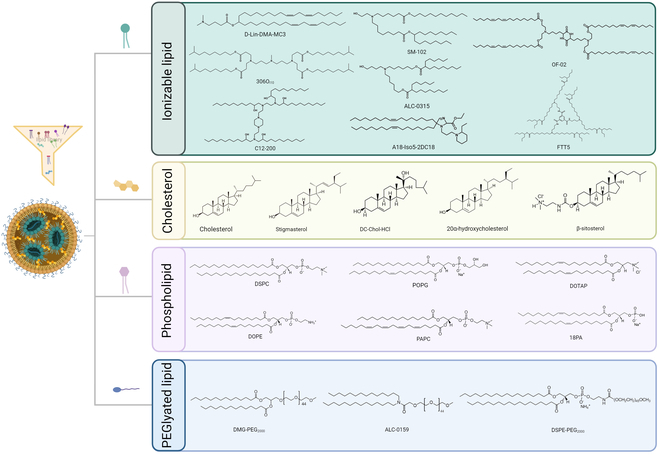
Examples of ionizable lipids, sterols, phospholipids, and PEG lipids used in LNP formulations. Created with BioRender.com.

Despite that researchers have most recently reported that iPLX phospholipid-free LNP systems presented exceptional stability [28], superior mRNA encapsulation efficiency, and sustained robust delivery efficacy, the three FDA-approved LNP-RNA products as well as most LNP-mRNA in preclinical reports conventionally are constituted of four components: ionizable or cationic lipids, sterols, helper lipids, and PEGylated lipids. To better illustrate the conceptions, the lipids mentioned in this review were demonstrated in Fig. 2. Cationic or ionizable lipids, such as 1,2-dioleoyl-3-trimethylammonium propane (DOTAP) or clinically approved DLin-MC3-DMA, primarily interact with negatively charged mRNA backbone to form electrostatic bonds, thus stabilizing the particles with enhancing encapsulation efficiency as well as the transduction efficacy [17,26,29,30]. Sterols, mostly present as cholesterol, are in charge of securing the particle's formation as a building block supplement. The cholesterol also shows the potential of enabling cellular uptake via ApoE-binding and low-density lipoprotein (LDL) receptor-mediated endocytosis [31,32]. Helper lipids, resembling the cell membrane bilayers, maintain LNP stability by filling the particles with hydrophobic lipid tails [33,34]. PEG-anchored lipids regulate particle size by controlling the fusion rate of vesicles during LNP formation while playing a substantial role in enhancing the in vivo bioavailability and biocompatibility, pharmacodynamics, and pharmacology [35–37].

### Ionizable cationic lipid

Ionizable cationic lipids play the most important role in determining the capability of LNPs, such as cytosolic transport and endosomal escape, for RNA to be released and functionalized. DLin-MC3-DMA (MC3), known for its strong efficacy in assisting RNA interference (RNAi) gene silencing in the liver, has been approved as the first siRNA drug, Onpattro, by the FDA in 2018 [26]. This particular lipid was discovered and optimized with a high-throughput library selection method. The early-stage library screening, initiated by Akinc et al. [38], involved the development of a lipid-like library via click chemistry, enabling the efficient synthesis of structurally diverse lipidoids in a short time frame. This library encompassed over 1,200 lipidoids that underwent screening on HeLa cells in vitro and paved the beginning road for discovering the MC3 lipid. This method offers the high-throughput selection of beneficial lipid structures for RNAi therapy and later encouraged many related findings with successful translation for the ionizable lipid screening for mRNA therapeutics [39–41]. In the perspective of chemistry, ionizable lipids could be divided into three parts: head, linker, and hydrocarbon tail [42], and most lipids in high-throughput and combinatorial library screening were constructed based on the random combination of these three parts. The head group of the cationic or ionizable lipid typically contains amine, guanidine, and heterocyclic group, which contribute to the positive charges required for the electrostatic interactions with the negatively charged mRNA [15]. Specially, ionizable lipids with heterocyclic head groups have the potential for extrahepatic delivery. For example, the lipid C12-200 with an optimized formulation can achieve tissue-selective delivery in the spleen [43], and OF-Deg-Lin (OF-02) targeted lymphocytes and achieved over 85% transfection efficiency in total protein expression in the spleen [44]. The linker and tail of an ionizable lipid often influence the clearance, fluidity, and delivery efficiency and should be taken into consideration as well. The linker connects the hydrophilic head and hydrophobic tails and can be classified into two categories: biodegradable and nonbiodegradable. Common linkers include ether, ester, amide, urea, and carbamate [15].

**Fig. 3.**
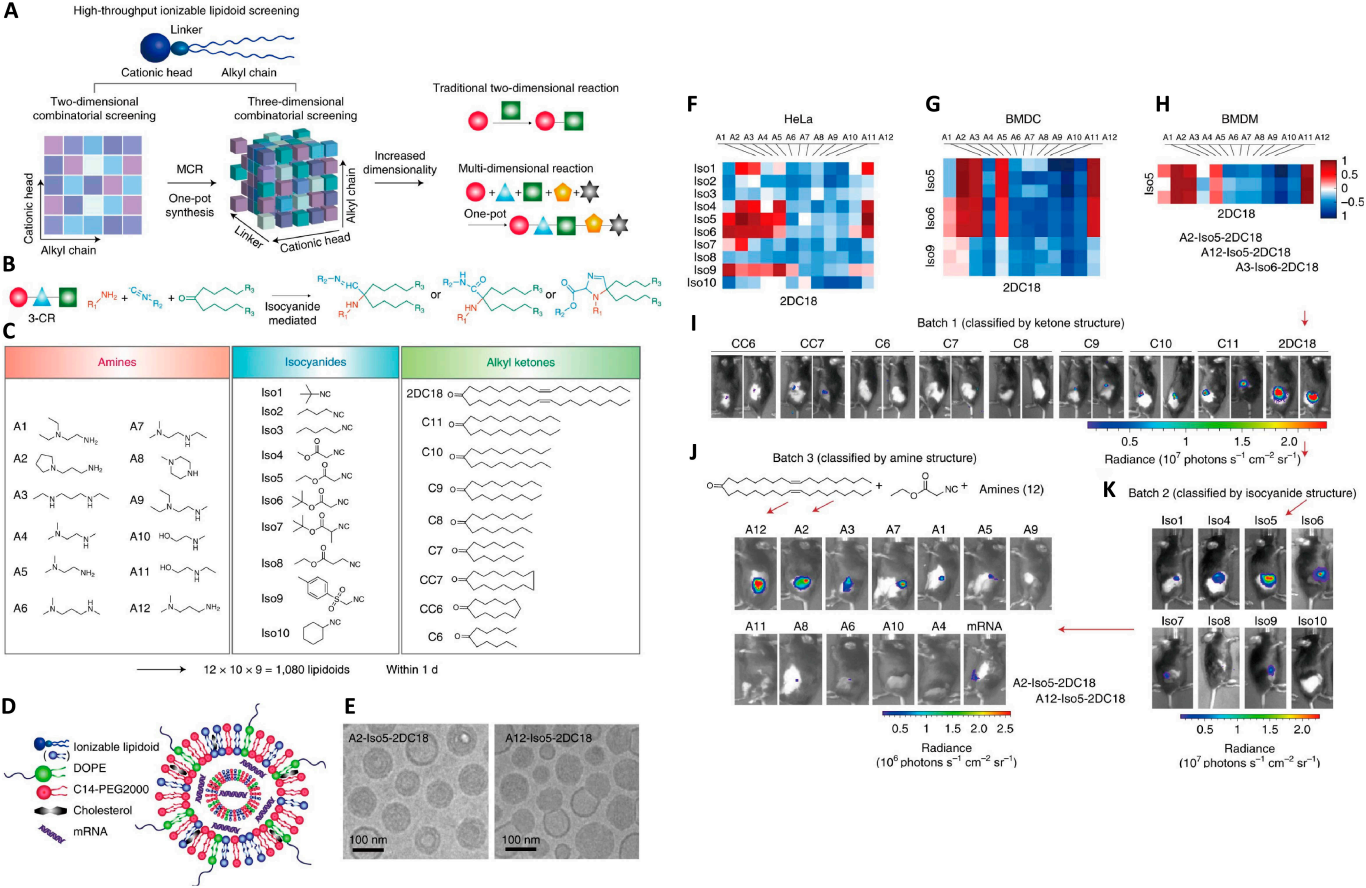
High-throughput screening of the three-component ionizable lipidoids facilitates the potent mRNA delivery in vitro and in vivo. (A to E) Schematic illustration of reaction mechanism and component structures of the screened lipidoids, along with representative LNP morphology via cryogenic electron microscopy images. (F to H) In vitro luciferase mRNA delivery efficacy among a range of 48 top performing lipids in HeLa cell line and mouse bone marrow-derived dendritic cells (BMDCs) and macrophages (BMDMs). (I to K) In vivo luciferase mRNA delivery for selecting optimal ketone, isocyanide, and amine structures, resulting in three top-performing lipids. Reproduced with permission [39]. Copyright 2019, Springer Nature.

In a most recent study illustrated in Fig. 3A to E, Anderson and Langer’s group has screened 1,080 ionizable lipidoids conjugated by different cationic head, linker, and alkyl tail chain for identifying top candidates that could induce a robust immune response for mRNA cancer vaccines [39]. They first identified 232 top-performing lipidoids and screened out top 2 lipids (A2-Iso5-2DC18 and A12-Iso5-2DC18) for their enhanced capability for mRNA delivery and expression efficacy in vitro and in vivo (representative results shown in Fig. 3F to K). By further analysis with a top lipid candidate, they demonstrated that lipidoid with heterocyclic cationic head group mediated significantly higher cytokine secretion at the injection site with more activated antigen-presenting cells (APCs) through the intracellular STING pathway, resulting in enhanced antitumor efficacy with limited systemic cytokine expression compared to Toll-like receptor activation, proving that cyclic lipid systems can be used for safe and efficacious antitumor immunity. The high-throughput method has also been applied by Siegwart’s research group for ionizable phospholipid screening to deliver mRNA and single-guide RNA (sgRNA) to facilitate gene-editing therapy in lung, spleen, and liver, respectively [45].

High-throughput screening method for ionizable lipid selection has proved efficacious, while massive effort and resource waste needed for in vivo selection still encounters significant challenges. Therefore, ionizable lipids could also be discovered and selected within a delicate library for limited structures. Ni et al. [46] synthesized and characterized 128 piperazine-based lipids using a DNA barcode high-throughput screening system. The best-performing lipid-formulated LNP-A10 delivered mRNA encoding Cre recombinase in immune cells in vivo at a dosage as low as 0.3 mg/kg, resulting in 50% tdtomato^+^ Kupffer cells, 23% tdtomato^+^ splenic macrophages, and 26% tdtomato^+^ splenic dendritic cells. Mitchell’s group from Pennsylvania University identified ionizable lipid with heterocyclic head from a library of 24 lipids and demonstrated its potential for mRNA delivery for human chimeric antigen receptor (CAR) T cell engineering. The purified C14-4 LNPs in vitro delivered CD19-CAR-encoded mRNA to T cells and caused high-level expressions of CAR molecules on the cell surface. After the adoptive transfer of the ex vivo priming T cells, the evident antitumor effect was observed [47]. The TT3 lipid was also developed within a small library ranging from TT2 to TT8 [48] and now has been applied for mRNA delivery in various applications including cancer immunotherapy [49] and vaccines [50]; the optimized version FTT5 lipid has been applied for genome editing applications [51]. Eygeris et al. [52] have rationally designed 47 ionizable lipids based on thiophene moiety as the core of the head group. Among them, lipid 20b emerged as the most potent lipid, effectively delivering Fluc-mRNA to the liver and spleen upon intravenous administration in mice. They also identified another lipid 29d for its extrahepatic organ selective capability and attributed its lung and spleen tropism for its zwitterionic nature. Unexpectedly, they found that lipid 20b-containing LNPs could deliver mRNA to photoreceptor cells after subretinal injection in both mice and nonhuman primates (NHPs), with no observed safety concerns. These findings suggest that their screened lipids hold promise for mediating LNP-mRNA-based therapy for pulmonary and retinal genetic diseases. Researchers paid less attention to investigating the linker and tail chain groups for ionizable lipid modification. Commonly, biodegradable linkers and tails are preferred in the design of novel lipids due to their lower cytotoxicity and rapid in vivo clearance, resulting in minimized side effects. Interestingly, Xu’s group [53] has discovered that a slight change in the linker group from ester to amide bond shifted the LNP delivery preference from liver to lung after intravenous administration, followed by the hypothesized explanation for the different corona protein binding for different ionizable lipids. On the other hand, first-generation lipids such as MC3 and KC2 have two unsaturated linoleic acid tails that facilitate membrane fusion in vivo due to their cone-shaped geometry when formulating LNPs. Branched tails also exhibit unique properties in terms of ionizable lipid performance. For instance, 306Oi series lipids demonstrated significantly higher protein expression in the liver compared to C12-200 and MC3 [54].

Though conceptionally via rational design, the discovery of ionizable lipid structure largely and realistically relies on random structural combinations of head, linker, and carbon chain tail, followed by the optimal lipid identification by analyzing the in vitro and in vivo mRNA delivery efficacy. There remains limited research conducted and few explanations to clarify how the structure of lipids affects the delivery activity of the LNP-mRNA. As a result, there is no clear theoretical basis to guide researchers to develop LNP for targeting respective organ of interest for mRNA medicines via certain administration routes to treat specific conditions. Further effort should be attempted at both academic institutes and industrial pharmaceutical companies to promote authentically rational design for the ionizable lipids with deep exploration and understanding the structure–activity relationship, which will largely benefit the development of LNP-mRNA therapeutics.

### Phospholipid and cholesterol

**Fig. 4.**
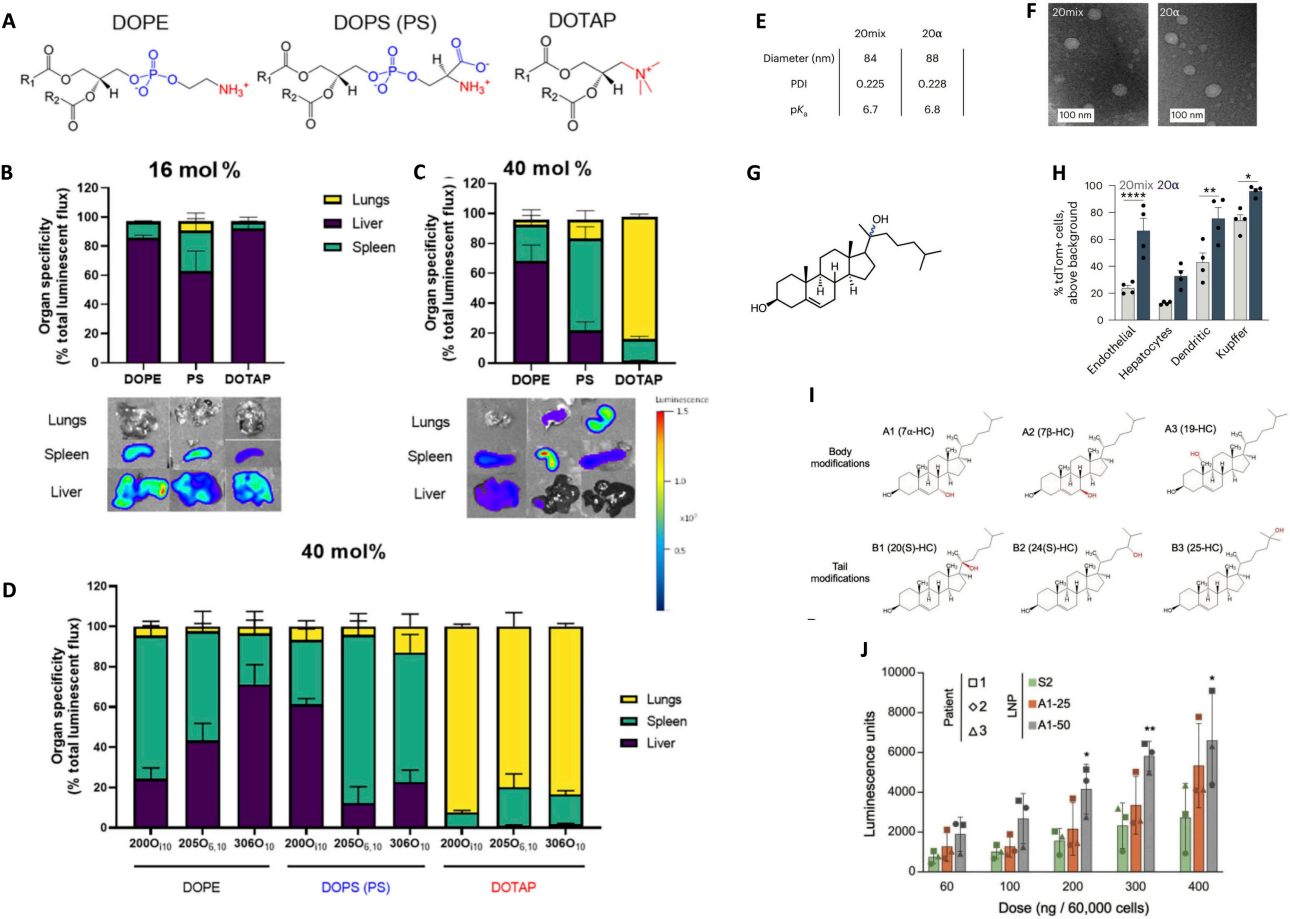
The effect of phosphate lipids (A to D) and cholesterol (E to J) on LNP-mRNA targeting and delivery efficacy. (A to D) Targeted mRNA delivery into specific organs (lung, spleen, and liver) via charged phospholipid-incorporated LNPs. Reproduced with permission [56]. Copyright 2022, Elsevier. LNPs were prepared using one of three helper lipids [chemical structure shown in (A)]: DOPE (with a net neutral charge), phosphatidylserine (PS) (with a net negative charge), or DOTAP (with a positive charge). (B) Ex vivo images demonstrated that luciferase expression occurred predominantly in the liver, regardless of helper lipid charge, for a standard LNP formulation (16 mol % helper lipid) incorporating the ionizable lipidoid 306O_10_ (0.75 mg kg^−1^ Luc mRNA, intravenous administration, 3 h). (C) Ex vivo images demonstrated that luciferase expression shifted from the liver to the spleen or lungs when LNPs were formulated with 40 mol % PS or DOTAP instead of DOPE (0.75 mg kg^−1^ Luc mRNA, intravenous administration, 3 h). (D) Quantitative luciferase signal from In Vivo Imaging System that presented the charge of the helper lipid influenced the organ site of protein expression regardless of the identity of the ionizable lipidoid (200O_i10_, 205O_6,10_, and 306O_10_) in LNPs. (E to H) Enhanced mRNA delivery in hepatic cells with altered stereochemistry of cholesterol. Reproduced with permission [59]. Copyright 2023, Springer Nature. (E and F) Size, polydispersity index (PDI), pKa, and morphology characterization of LNPs formulated with 20α or 20mix. (G) 20-Hydroxycholesterol chemical structure, with stereocenter highlighted in dark blue. (H) The 20α LNPs facilitated significantly higher liver-targeted delivery among all types of cells compared to 20mix LNPs, as shown by the percentage of tdTom^+^ cells, measured by flow cytometry, 3 days after Ai14 mice were injected with 0.3 mg/kg (body weight) of LNPs formulated with 20α or 20mix. (I and J) LNP formulated with hydroxycholesterol substitutes mediated higher mRNA delivery to human T cells. Reproduced with permission [60]. Copyright 2022, Elsevier. (I) Chemical structures of the hydroxycholesterol substitutes. (J) LNPs formulated with substitution of 7α -hydroxycholesterol by 25% (A1–25) and 50% (A1–50) assisted higher primary human T cells compared to standard LNP formulation (S2) at various doses, characterized by the luciferase mRNA expression.

Phospholipids play a crucial role in the design and function of LNPs as a structural component and are significant for LNP in targeted delivery of therapeutic molecules, such as mRNA and gene-editing tools. The three FDA-approved LNP-mRNA products all applied 1,2-distearoyl-sn-glycero-3-phosphocholine (DSPC) as their helper lipid; researchers also utilize novel phospholipids such as 1,2-dioleoyl-snglycero-3-phosphoethanolamine (DOPE) for extrahepatic targeting site and enhanced delivery efficacy. Kuninty et al. [55] developed tail-flipping nanoliposomes engineered to target alternatively activated macrophages for cancer immunotherapy. The researchers employed phospholipids to formulate LNPs to achieve selective targeting of macrophages. By utilizing specific phospholipid 1-palmitoyl-2-azelaoylsn-glycero-3-phosphocholine (PAPC), they were able to enhance the therapeutic efficacy of the nanoliposomes and facilitate targeted delivery to cancer-associated M2 macrophages. The replacement of neutral DOPE lipid for charged lipids 1,2-dioleoyl-sn-glycero-3-phospho-l-serine (DOPS), 1,2-dioleoyl-sn-glycero-3-phosphate (DOPA, 18PA), and DOTAP mainly shifted liver-oriented LNP to lung and spleen, respectively (Fig. 4A to D). Especially, when the ratio of phospholipid increased from 16% to 40%, complete replacement of DOPE with cationic DOTAP to a great extent mediated LNP to deliver mRNA to the lung, whereas negatively charged DOPA-inserted LNP mainly delivered mRNA to the spleen [56].

Cholesterol (CHO), as a naturally abundant sterol lipid, is essential in the building blocks of LNPs and maintains the stability of LNP structure. Due to its small molecule size, CHO can fill the cavity within the LNPs, reduce the leakage of the nuclear acids, and increase the integrity of the nanoparticles. The molar ratio of CHO in the LNP formation varies from 20% to 50%, yet its significance in LNP formation and function cannot be dismissed. Completely replacing CHO with an alkyl substitute resulted in morphology and structure change. Patel et al. [57] discovered that without cholesterol insertion, LNP formed a nonlamellar structure and resulted in a low encapsulation efficiency. The changes in morphology not only altered mRNA packing but affected the efficacy of cellular uptake and the intercellular trafficking of LNPs. According to Kawaguchi et al. [58], the cellular internalization and protein expression decreased both in vitro and in vivo when the molar percentage of CHO was reduced from 40% to 10%. In addition, at a lower CHO content, the size of LNPs increased from 75.4 nm to 140 nm, and encapsulation efficiency decreased to 65%, causing unstable physicochemical properties of LNPs. Additionally, CHO can be chemically modified to increase the delivery efficiency of LNPs. Hatit et al. [59] discovered and deciphered the importance of nanoparticle stereochemistry in altering and enhancing the endocytic process. They compared different types of hydroxycholesterol and formulated LNPs consisting of pure 20α-hydroxycholesterol (20α) and cholesterol mixture containing both 20α-hydroxycholesterol and 20β-hydroxycholesterol (20mix). They found that 20α-hydroxycholesterol significantly improved in vivo hepatic delivery efficacy and cellular expression of mRNA in different types of liver cells (Fig. 4E to H). Similarly shown in Fig. 4I and J, substituting cholesterol with hydroxycholesterol by a 50% ratio also demonstrated that primary human T cells resulted in twofold higher transfection efficacy, providing a potential mRNA treatment for immunotherapies [60]. Herrera et al. [61] investigated five different cholesterol analogs, in which β-sitosterol and stigmasterol-substituted LNPs showed multi-faceted architectures compared to standard cholesterol-containing LNP and resulted in a 10-fold increase in endosomal perturbation efficiency.

**Fig. 5.**
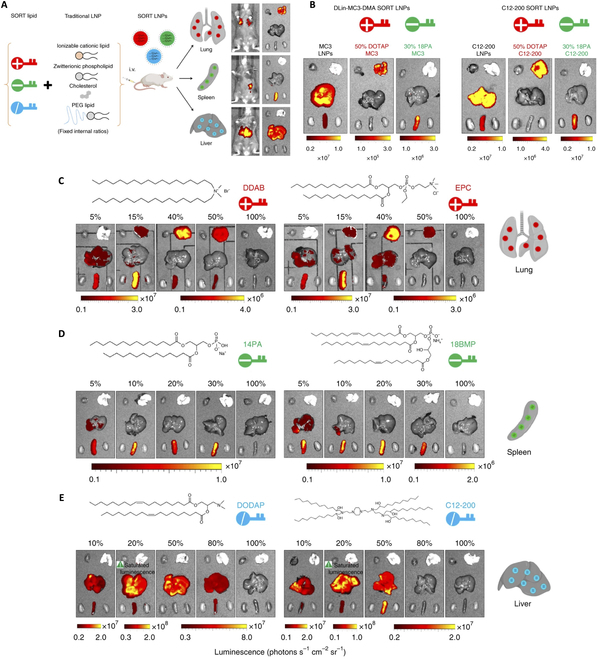
Selective organ targeting (SORT) enables the systematic and reliable engineering of lipid nanoparticles (LNPs) for precise mRNA delivery into specific organ. Reproduced with permission [62]. Copyright 2020, Springer Nature. (A) When a supplementary component, referred to as a SORT molecule, is introduced to conventional LNPs, it systematically modifies the in vivo delivery profile, facilitating tissue-specific delivery based on the percentage and biophysical properties of the SORT molecule. This strategy effectively redirected various categories of nanoparticles. (B) Ex vivo images demonstrate luminescence in major organs using DLin-MC3-DMA SORT LNPs and C12-200 SORT LNPs, incorporating cationic lipid (DOTAP) and anionic lipid (18PA) in the formulations (0.1 mg kg^−1^ Luc mRNA, intravenous administration, 6 h). (C to E) SORT molecules could be divided into specific groups with defined biophysical properties despite distinct chemical structures. (C) Permanently cationic SORT lipids (dimethyldioctadecylammonium (bromide salt) （DDAB）, 1,2-dioleoyl-sn-glycero-3-ethylphosphocholine (EPC), and DOTAP) all resulted in the same mRNA delivery profile to the lung. (D) Anionic SORT lipids (14PA, 18BMP, and 18PA) all resulted in the same mRNA delivery profile to the spleen. (E) Ionizable cationic SORT lipids with tertiary amino groups (DODAP, C12-200) enhanced liver delivery without luciferase expression in the lungs (0.1 mg kg^−1^ Luc mRNA, intravenous administration, 6 h).

Besides the incorporation of cholesterol derivatives and innovative phospholipids, researchers also aim to modulate the ratios among the lipid components and realize targeted delivery of mRNA to the specific organ with improved efficacy. Zhang et al. [43] utilized in vivo library screening containing 96 LNP formulations with different ratio of lipids. They identified a specific LNPs formulation consisting of DSPC substantially accumulated in the spleen, while identical LNPs formulations with substitution of DOPE preferentially distributed in the liver. The insertion of a “fifth” lipid component poses as additional strategy for manipulating targeted delivery via LNP-mRNA. In a study by Dilliard and colleagues [62,63], the authors demonstrated that LNPs composed of additional phospholipids with specific biophysical property—electrostatic charge—could efficiently deliver mRNA to target specific tissues (Fig. 5A), naming the LNPs selective organ targeting (SORT) nanoparticles. For instance, in addition to conventional four lipid components, with positively charged SORT lipid DOTAP added, LNPs facilitated targeted delivery of mRNA to the lung, whereas additional negatively charged SORT lipid, 18PA, assisted LNPs to achieve spleen targeting (Fig. 5B). They further discovered that in spite of the different chemical structure, SORT lipids holding similar biophysical property possessed the same ability to assist LNP to enable targeting delivery of mRNA. For instance, they examined two anionic helper lipids with distinct chemical structure, 1,2-dimyristoyl-snglycero-3-phosphate (14PA) and sn-(3-oleoyl-2-hydroxy)-glycerol1-phospho-sn-3′-(1′,2′-dioleoyl)-glycerol (18BMP). All the anionic SORT lipids promoted exclusive delivery to the spleen (Fig. 5D).

The findings suggest that the SORT methodology offers versatility in selecting molecules, paving the way for optimizing future SORT molecules to achieve a balance between potency, selectivity, and tolerability. The identification of SORT, enabling predictable nanoparticle delivery of RNA to specific organs, is expected to significantly contribute to the advancement of protein replacement and gene correction therapeutics.

In summary, the specific stereochemical configuration of cholesterol and its derivatives exhibited a profound impact on morphology, structure, size, and encapsulation efficiency, as well as the potential function of LNPs in facilitating mRNA delivery. The phospholipids additionally play a crucial role in the design and performance of LNPs for targeted drug delivery, affecting the biodistribution, tissue-specific delivery, and therapeutic efficacy of LNPs. The molar ratio among lipid components is also vital for organ-specific delivery and worthy for explorations. Despite the wide preclinical investigations of SORT LNPs to deliver mRNA to facilitate protein replacement or genome editing, clinical translation needs further thorough consideration. The in vivo safety and degradation of the fifth component remain problematic, whereas the on-shelf process development and quality control for the SORT LNPs pose as a difficult issue. Therefore, although innovative and insightful, the SORT LNPs, as all the breakthrough inventions in targeted delivery technology, may invest more efforts in paving the way toward clinical translation to expand and broaden the therapeutic range for mRNA medicines.

## Strategies for Enhanced Targeting Delivery and Efficacy

**Fig. 6.**
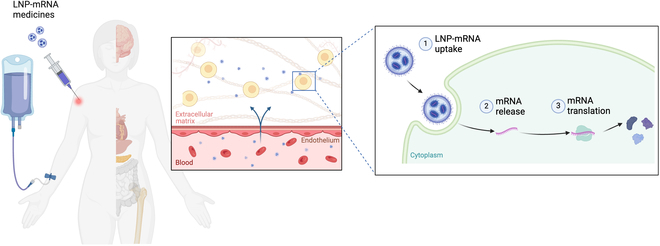
Schematic illustration of in vivo fate of LNP-mRNA modality after administration into the body, including encompassing systemic circulation, cellular uptake, mRNA release into the cytosol, and subsequent translation into proteins. Created by Biorender.com.

As forementioned, the manipulation and optimization of ionizable lipids, phospholipids, and cholesterol have to some extent enabled targeted delivery of mRNA to specific organ such as liver, lung, and spleen. Other than lipid structure, surface modification, and LNP formulations critically affect mRNA loading, targeted delivery, and therapeutic efficacy, the relevant scientific discovery has been intensively reviewed in this section. Figure 6 illustrates the in vivo fate of the LNP-mRNA modality after administration, encompassing systemic circulation, cellular uptake, mRNA release into the cytosol, and subsequent translation into proteins. In addition, we provide critical opinions regarding the innovative LNP screening approaches and unmet needs for the LNP strategies in mRNA medicine development.

### Surface modification of LNPs

#### PEGylated lipid—Modulating serum protein adsorption for tissue targeting

Pegylation of LNP surface via polyethylene glycol (PEG) plays a pivotal role in improving LNP stability and facilitating readily tissue penetration and targeting. Pegylation strategy has been widely used in many drug modalities and delivery systems. Since PEG is an amphiphilic lipid that can interact with both the hydrophobic core of the LNPs and the hydrophilic environment of physiological conditions or storage buffers, it plays a crucial role in regulating the clearance and half-life of LNPs, as well as on-shelf stability. Typically, the half-life of LNPs depends on the length of PEG lipid. The longer the PEG length, the less easily nonspecific interaction with serum components in the bloodstream. Nonetheless, it also inhibits fusion between LNP and endosome membrane, widely known as the “PEG dilemma” in LNP drug delivery [64]. The lipid structure of pegylation also has a significant effect on LNP function.1,2-dimyristoyl-rac-glycero-3-methoxypolyethylene glycol-2000（DMG-PEG_2000_), 1,2-distearoyl-sn-glycero-3-phosphoethanolamine-N-[amino(polyethylene glycol)-2000] （DSPE-PEG_2000_）, and 1,2-dimyristoyl-sn-glycero-3-phosphoethanolamine-N-[methoxy(polyethylene glycol)-2000] (14:0 PE-PEG_2000_) have been widely applied in LNP formation, commonly leading to different delivery efficacy. PEG lipid with shorter anchor such as DMG or C14 resulted in quick desorption from LNPs once in circulation [35], while PEG lipids with longer C18 alkyl chain were more capable of longer circulation [65]. In a nonviral gene therapy to treat traumatic brain injury, DMG-PEG_2000_ and DSPE-PEG_2000_ were compared. They formulated siRNA and mRNA LNPs with various ratios of DMG-PEG_2000_ and DSPE-PEG_2000_ and discovered that the increase in the proportion of DSPE-PEG_2000_ led to longer blood circulation with enhanced gene expression in the injured brain hemisphere [66].

Besides optimizing the choice of the hydrophobic domain of PEG lipids, regulating the affinity (or shedding rate) of PEG lipids is another trending strategy. The presence of PEG on the surface of nanoparticles may hinder cell uptake of nanoparticles. PEG lipids on LNPs are expected to shed from LNP in a controlled way after administrations, thus inducing cellular internalizations at the target site and also maintaining high serum stability. Suzuki et al. [67] revealed the influence of PEG shedding rate on the blood clearance of PEGylated LNPs. Specifically, faster PEG shedding led to attenuated production of anti-PEG immunoglobulin M (IgM), indicating that optimization of the kinetics of PEG shedding can improve the circulation and biodistribution of LNPs in the body. Moreover, the pharmacokinetics and pharmacodynamics of siRNA-loaded LNPs could be influenced by the desorption rate of PEG. It is essential to understand the kinetics of PEG dissociation, as it directly impacts the duration and effectiveness of the therapeutic payload in target tissues. Mui et al. [35] first investigated the gene knockdown efficiency associated with PEG length and PEG content within LNPs. Their group found that hydrophobic interactions with cell membranes were proportional to the PEG alkyl length. Specifically, when fixing PEG content at 1.5%, C16 and C18 PEG need more time to shed from the surface of LNPs compared to C14 PEG. When increasing the PEG content from 1.5% to 3%, C14 PEG LNPs showed a gradual decline in gene knockdown, while C18 PEG completely lost its function when its content was higher than 1.5%. This phenomenon can be attributed to the longer alkyl length of C18 PEG, which increased the hydrophobicity of PEG lipid, and hindered the interaction with the cell membrane. As a result, longer alkyl length required more energy for LNPs to adopt a monolayer transition state, leading to shedding of PEG from the LNP surface. Thus, C18 PEG has a longer circulation time after administration but still holds its ability with 1.5% threshold concentration.

In another aspect, PEG shedding is due to the pH change during the endocytosis process. A study by Hashiba and colleagues [68] demonstrated the benefits of pH-labile PEGylation in siRNA-loaded LNPs for hepatocyte targeting and gene silencing activity. The study highlighted that pH-responsive PEG lipids facilitated improved active targeting and gene silencing efficacy in hepatocytes. Furthermore, the PEG content significantly affects targeting of extrahepatic tissues. For instance, Zhang et al. [69] engineered aerosolized LNPs for pulmonary delivery of mRNA. They optimized PEGylation parameters to achieve efficient mRNA delivery to the lungs. They found that increasing the PEG ratio in the LNP formulation not only reduced the size of LNPs but also decreased intracellular protein expression of mRNA. Therefore, precise consideration of the extent of PEG-anchored lipids is crucial when designing LNP-mRNA for lung therapeutics. This approach represents a significant advancement in developing noninvasive respiratory therapies with potential applications in treating various pulmonary diseases.

The protein corona phenomenon, mainly influenced by the surface properties of LNPs, has become popular among researchers in the field of nanosized drug delivery systems. After the administration of LNPs, serum proteins are adsorbed on the surface of the nanoparticles evidently, forming a protein corona. This corona may impede targeted efficacy by shielding surface ligands necessary for specific cell or receptor recognition. Surface-exposed PEGylated lipids play a crucial role in determining and regulating the composition of the corona proteins on LNPs. Parallelly, the compositions of the corona proteins can govern the distributions of the nanoparticles due to the organ-specific accumulations of the serum proteins, such as ApoE for the liver and complementary system-related proteins for the spleen.

In a recent study, Chen et al. [70] investigated the role of apolipoprotein- and vitronectin-enriched corona on LNPs for targeted delivery and transfection of oligonucleotides in murine tumor models. A slight change in the physicochemical properties of LNPs altered the corona pattern. For instance, the introduction of positive charge via cationic lipid 3β-{N-[2-(dimethylamino)ethyl]carbamoyl}cholesterol （DC-cholesterol） as a substitution for original cholesterol shifted the protein corona pattern from apolipoprotein-rich to vitronectin-rich. In the aspect of transfection performance, nanoparticles with apolipoprotein-rich corona showed better delivery to hepatocellular carcinoma (HepG2) as compared to those with vitronectin-rich corona. LNPs formulated with PEG-conjugated C18 lipids (either 3% or 6% molar ratio) were proved to be optimal for in vivo delivery to HepG2 tumor. The study revealed that the composition of the protein corona influenced the biodistribution and targeting efficiency of LNPs, highlighting the complex interplay between nanoparticle surface properties, including surface-coating PEG, protein interactions, and in vivo performance.

In conclusion, PEGylated lipids play multiple roles in enhancing the performance of LNPs as drug delivery systems for therapeutic mRNA. Through studies that examined PEG shedding rates, protein corona formation, and pH-labile PEGylation, researchers have gained valuable insights into optimizing the effectiveness of LNPs by manipulating surface PEG. These advancements in PEGylation strategies hold promise for the design of precise and efficient delivery systems for therapeutic nucleic acid, especially mRNA.

#### Antibody modifications for ligand–receptor-mediated tissue targeting

**Fig. 7.**
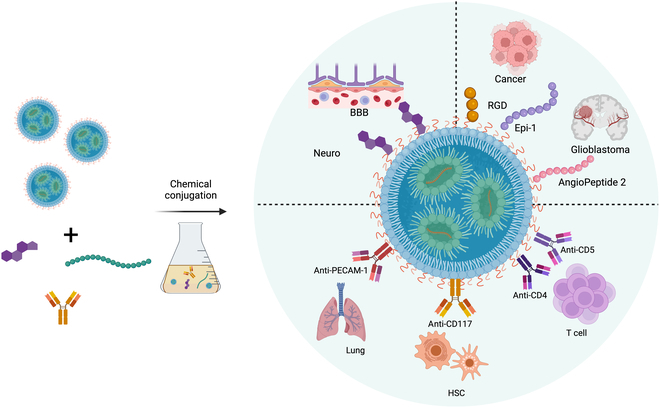
Surface modification strategies for tissue targeting of LNP-mRNA. Created with BioRender.com.

To develop effective targeting strategies, it is crucial to enhance therapeutic potential in various diseases, especially those related to extrahepatic organs such as the brain [71], immune system [72], and cardiac tissues [73]. While LNPs have shown promise in assisting mRNA to treat a specific range of conditions, some of which are also under clinical and preclinical investigation, mRNA as a drug modality stays utilized in vaccines and in mainly treating liver disorders. Shortly, LNP-mRNA medicines for specific tissue disease intervention remain challenging. Hence, broadening the horizon of LNP-mRNA therapy into a wider range, such as in vivo CAR T therapy, antitumor therapy, gene editing to treat pulmonary fibrosis, amaurosis, Angelman syndrome, and thalassemia, largely relies on advancing technologies for specific tissue targeting. In most cases, the delivery vehicles depend on passive transport, wherein the circulating nanoparticles are taken up by tissues or cells, generally causing undesirably inadequate therapeutic efficacy and the side effects that caused by off-target distribution. A more precise targeting strategy is expected. To address this, one approach is to modify the surface of LNPs with active targeting ligands, especially antibodies, to interact with specific cell receptors (Fig. 7). In this section, preclinical applications of active targeting scheme of LNP-mRNA were detailed.

**Fig. 8.**
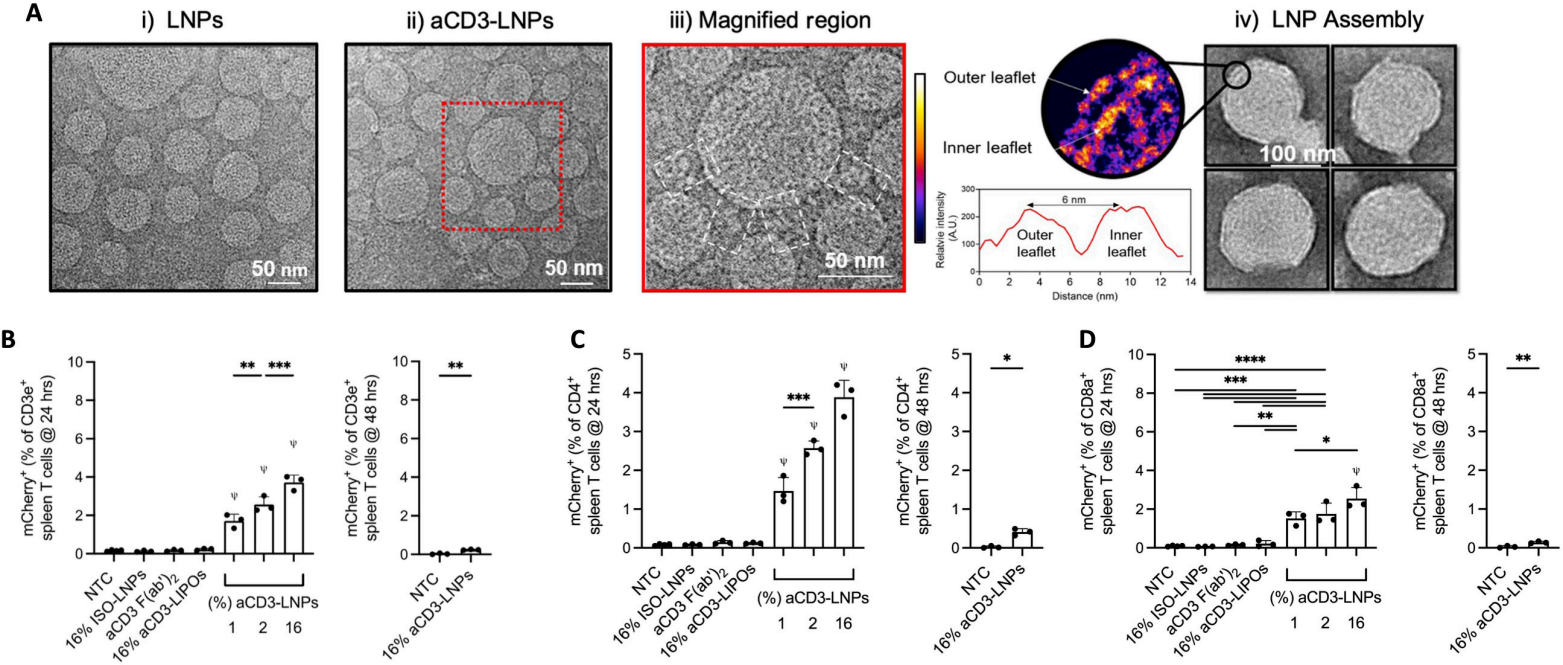
The characterization and in vivo T cell targeting delivery efficacy of anti-CD3 (aCD3)-conjugated LNP [75]. (A) Cryo-TEM images of the nontargeted LNPs and aCD3-conjugated LNPs with magnified regions in which white rectangles presented the conjugated 16% aCD3 on the surface of LNPs. (B to D) Delivery efficiency in vivo analyzed by mCherry-mRNA expression in the splenic CD3e^+^ (B), CD4^+^ (C), and CD8a^+^ T cells (D) at 24 and 48 h after control [no treatment, isotype-conjugated LNPs, or aCD3 F(ab′)2] or 1, 2, or 16% aCD3-LNP treatment, respectively.

Among all the extrahepatic organs, the spleen occupies one of the most favorable and intriguing targets. On the one hand, the spleen is a pivotal organ in the immune system, governing the priming and maintenance of various immunocytes, such as T cells, B cells, and APCs. On the other hand, the diversified immunocytes harbor distinct properties, for example, the enzyme enrichment of the granulocyte and the active phagocytosis of macrophages. As a major immunocyte in antitumor and antiviral therapy, T cells have attracted accumulating attention, yet are notoriously difficult to be transfected. Zhao et al. [74] conducted T cell modifications in vivo, which explored imidazole-based synthetic lipidoids to deliver mRNA to the T lymphocytes. After intravenous injection of LNPs carrying Cre recombinase mRNA, 8.2% of CD4^+^ T cells and 6.5% of CD8^+^ T cells were transfected, validated via flow cytometry, suggesting the potential of LNP-mRNA in facilitating in vivo spleen and T cell therapy. To further increase the targeting efficiency, antibody conjugation was introduced to enhance the uptake and transfection efficiency of T cells. Kheirolomoom et al. [75] conducted an innovative approach for in situ T cell transfection by employing anti-CD3-conjugated LNPs. They formulated LNPs with DLin-MC3-DMA lipid with cholesterol, DSPC, DSPE-PEG_2000_, and maleimide-DSPE-PEG_5000_ (in a ratio of 50, 38, 10, 1.5, and 0.5) in which maleimide group was for the conjugation of N-hydroxysuccinimide (NHS) activated CD3 antibody onto the LNP surface. As shown in Fig. 8A, the formulated LNPs were covered with CD3 antibody at a ratio of 16% observed and calculated via the cryo-transmission electron microscope (TEM) technique. In Fig. 8B to D, as opposed to no treatment control or nonconjugated LNPs in which groups failed to elicit comparable transfection efficacy, CD3-LNPs demonstrated transfection efficiencies of 4% mCherry mRNA expressing splenic CD3e^+^ T cell, 4% CD4^+^ T cell, and 2.5% CD8a^+^ T cell at 24 h after intravenous injection.

In a recent work, Tombácz et al. [76] from Acuitas Therapeutics achieved highly efficient CD4^+^ T cell targeting and genetic recombination using engineered CD4^+^ cell-homing mRNA-LNPs. They prepared LNPs with ALC-0307 ionizable lipid (proprietary to Acuitas Therapeutics), with CD4 antibodies conjugated onto the particle surface. Briefly, a sulfhydryl group was introduced to monoclonal CD4 antibodies via N-succinimidyl-S-acetylthioacetate (SATA) activation reaction. Then, SATA was deprotected by 0.5 M hydroxylamine solution. Finally, LNPs modified with DSPE-PEG-maleimide were covalently bonded with activated CD4 antibody. In their experiments, mice receiving injections of CD4-LNPs encapsulating luciferase mRNA exhibited a 7- and 33-fold increase in luminescence signal within the spleen and CD3^+^ T cells compared to mice injected with LNPs modified with control IgG antibodies. Furthermore, 24 h after intravenously injecting CD4-LNPs loaded with mRNA encoding Cre recombinase into Cre reporter mice, approximately 50% of CD3^+^CD8^−^ splenic T cells and 20% to 40% of CD3^+^CD8^−^ T cells in lymph nodes exhibited successful transfection. This study sheds light on the potential of precision medicine in immunotherapy via LNP-mRNA, where specific targeting is paramount for LNP delivery system to realize the successful modulation of immune responses via mRNA modality.

Beyond solely facilitating tissue targeting, conjugating antibody onto the LNP holds the potential to enable new realm of manufacturing process for mRNA-mediated T cell therapy. CAR T cell therapy has achieved remarkable clinical success in treating hematologic malignancies. Unlike traditional viral vectors to produce CAR T cells, mRNA does not integrate into the genome, potentially allowing for only transient CAR expression, which could help minimize durable adverse effects like cytokine release syndrome [77]. Additionally, nonviral delivery methods could reduce manufacturing costs and time, increase cargo capacity, and improve safety [78]. Recently, Mitchell’s group from Pennsylvania University, together with D. Weissman and C. June, enabled one-step production of mRNA CAR T cells via CD3/CD28 antibody fragment-conjugated LNP (aLNP) with the elimination of cumbersome steps of conventional magnetic bead activation of T cells [79]. The aLNPs efficiently transfected primary human T cells with luciferase mRNA in the absence of activating beads. With their optimal 1:10 aLNP, 82.7% of T cells were successfully transfected with CAR expression and facilitated 67.6% effective cancer cell killing. They observed that adoptive transfer of anti-CD19 CAR T cells generated with aLNPs reduced the tumor burden in xenograft mouse model of leukemia and extended survival of mice over phosphate-buffered saline (PBS) by 17 days, while treatment with lentiviral CAR T cells extended survival over PBS by 8 days. Their discoveries proved that aLNPs with surface-conjugated human CD3 and CD28 antibody fragments not only efficiently delivered CAR-encoding mRNA into T cells but also possessed the capability of activating beads. These CD3/CD28-conjugated LNPs hold promise for reducing the complexity, cost, and time associated with mRNA CAR T cell production. Moreover, this platform is well positioned to support more widespread immunotherapy applications.

**Fig. 9.**
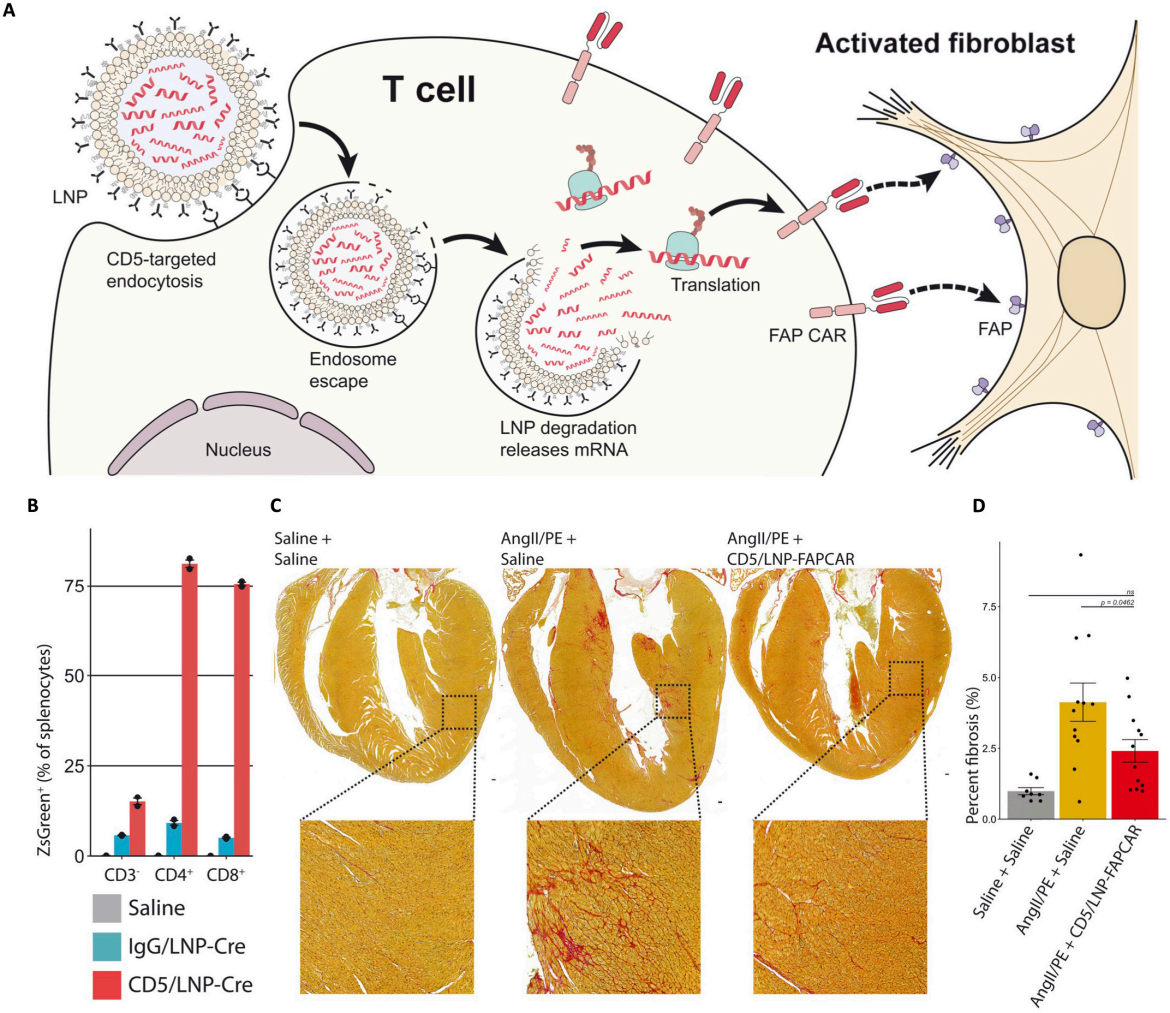
(A) Schematic illustration of CD5-targeted LNPs achieved in vivo production of CAR T cells. (B) Delivery efficiency demonstrated by Cre-mRNA expression among the splenocytes. Ai6 mice (Rosa26CAG-LSL-ZsGreen) were injected with either saline, IgG/LNP-Cre (30 mg), or CD5/LNP-Cre (30 mg). After 24 h, they observed ZsGreen expression in 81.1% of CD4^+^ splenocytes and in 75.6% of CD8^+^ splenocytes, whereas only 15.0% of CD3 splenocytes exhibited such expression. The bar graphs depict data from two independent biological replicates. (C and D) Images (C) and quantitative analysis (D) of fibrosis of ventricles in coronal cardiac sections of mock uninjured animals (3 weeks after saline pump implant + saline injection at week 1), injured control animals (AngII/PE + saline), and treated animals (AngII/PE + CD5/LNP-FAPCAR). Picrosirius red staining highlights collagen (pink). Reproduced with permission [80]. Copyright 2022, The American Association for the Advancement of Science.

In addition, antibody conjugation of LNPs expands beyond the realm of immunology. Rurik et al. [80] ventured into cardiac therapy by producing CAR T cells in vivo. As shown in Fig. 9A, the involvement of antibodies may increase the targeting efficacy but also compromise the T cell functions if selected vaguely. Here, they linked the LNPs with CD5 antibody instead of CD3 or CD4 antibody, employing similar conjugation procedures as Acuitas Therapeutics. The specificity of CD5-LNPs was evaluated by encapsulated mRNA encoding Cre-recombinase and validated in Cre-loxP reporter mice, and they discovered that 81.1% of splenic CD4^+^ T cells and 75.6% of splenic CD8^+^T cells exhibited reporter protein expression, indicating the high delivery efficiency (Fig. 9B). To prove the therapeutic effect of active targeting LNP-mRNA, the researchers extended their investigation to delivery of fibroblast activation protein-targeted CAR (FAPCAR)-expressing mRNA into T cells (CD5-LNP-FAPCAR, referring to anti-CD5 antibody-conjugated LNPs) in the context of cardiac injury and fibrosis. At 48 h after the injection of CD5-LNP-FAPCAR, a notable percentage (ranging from 17.5% to 24.7%) of FAPCAR^+^ T cells were identified in the spleens of the mice (Fig. 9C). Importantly, this intervention had a significant impact on cardiac function improvement in the injured mice, with observable benefits evidently at 2 weeks after injection of CD5-LNP-FAPCAR (Fig. 9D). This achievement underscored the potential of targeted LNPs for CAR T cell therapies in addressing cardiac fibrosis, offering a novel avenue for therapeutic advancements. Recently, a startup company, Capstan, has been founded based on the technique advancements by a group of scientists from Pennsylvania University, with hopes of applying active targeting LNP-mRNA therapeutics for interventions beyond oncology, but into more common and rare diseases.

Shi et al. [81] took a significant step forward by demonstrating in vivo RNA delivery to hematopoietic stem and progenitor cells using CD117-conjugated LNPs. Hematopoietic stem cells (HSCs) are crucial in maintaining the blood and immune systems, making them valuable therapeutic targets. By delivering CD117-conjugated LNP with Cre-recombinase mRNA, almost 90% of HSCs were genetically edited at a dose of 1 mg/kg (mpk) in vivo. The conjugation of antibodies to LNPs in this study represented a breakthrough in the field of regenerative medicine and opened new avenues for modifying these critical cell populations to treat hematological disorders. Similarly, Breda et al. [82] also used CD117 antibody-conjugated LNP delivery system to efficiently deliver mRNA to long-term HSCs (LT-HSCs) in the bone marrow niche. When delivering mRNA encoding Cre recombinase with single systemic administration of CD117-LNP, durable genome editing efficacy up to 4 months was achieved at a level of 55% in LT-HSCs (threefold as compared to the IgG-LNP group, 55% versus 19%), which has been reportedly required for the cure of nonmalignant hematopoietic disorders. This study also demonstrated that CD117-LNP delivering mRNA could be applied for HSC depletion prior to bone marrow engraftment without the genotoxic conditioning regimens that often result in pulmonary, liver, and reproductive toxicity. By intravenously injecting CD117-LNP-mRNA encoding PUMA (p53 up-regulated modulator of apoptosis) at 0.05 mpk in C57BL/6 mice, a 71% and 58% decrease in the frequency of Lin^−^Sca1^+^cKit^+^ (LSK) cells and LT-HSCs in bone marrow was shown at 6 days after treatments, respectively, demonstrating highly effective and safe approach for prior HSC depletion treatment for bone marrow engraftment therapy. In summary, by conjugating proper antibodies to LNPs, researchers can enhance the specificity of gene delivery to these stem cells, thereby improving the precision and safety of such interventions.

In conclusion, pegylated surface and ligand modification largely determine the biodistribution and in vivo fate of LNPs, thus affecting the delivery and therapeutic efficacy of loaded mRNA medicines. Modulation of the pegylated lipid alone may have limited impact on LNPs without clarified mechanism. While antibody modification might have provided solution by boosting the mRNA targeted delivery to specific cell type, this strategy remains in the preclinical stage for several reasons including limited antibody selection with low specificity, difficulties in process development, and quality control. Moreover, antibodies may harbor the potential to comprise the cell functions. Further efforts are expected to precisely control the conjugation density among LNPs to balance the targeting efficiency and biofunctions of target cells. Additionally, fostering new target ligands with high specificity and low toxicity is demanded. Small molecules and peptides serve as ligand alternatives for LNP to facilitate targeted delivery, including neurotransmitter for brain targeting [71], mannose for APC targeting [83,84], angiopeptide2 for glioblastoma targeting [85], and CPP peptide RGD (RRRRRRGGRRRRG) [86] and Epi-1 peptide (D-WRPTRURLLPWWICGSGSK) for tumor targeting [87]. Among the preliminary research, few have been applied on LNP for mRNA delivery. Another pivotal aspect is the in vivo fate of the surface-modified LNPs. Although several groups unveiled the parameters that may affect the biodistribution and degradation of pegylated LNP, the antibody-conjugated PEG shedding, protein corona absorption, tissue biodistribution, and degradation in the circulation remain obscure. In this case, no defined protocols could guide the rational design of ligand-conjugated LNPs to facilitate and enhance the targeted delivery of mRNA in specific organ and cell. In summary, additional and continuous endeavors are anticipated to fill the niche and provide LNP for precisely targeted delivery to assist mRNA therapeutics to treat specific disorders.

### Emerging technologies boosting the tissue targeting efficacy

Researchers made substantial progress in multiple disciplines to smooth the pathway for mRNA therapeutics, especially in the advances of LNPs. Along with these achievements reached in material science and surface modification, scientists have developed approaches for boosting the delivery efficacy of LNP. These advancements provide insights into the principles guiding the design and optimization of LNP delivery systems for a broader spectrum of mRNA therapeutic applications.

**Fig. 10.**
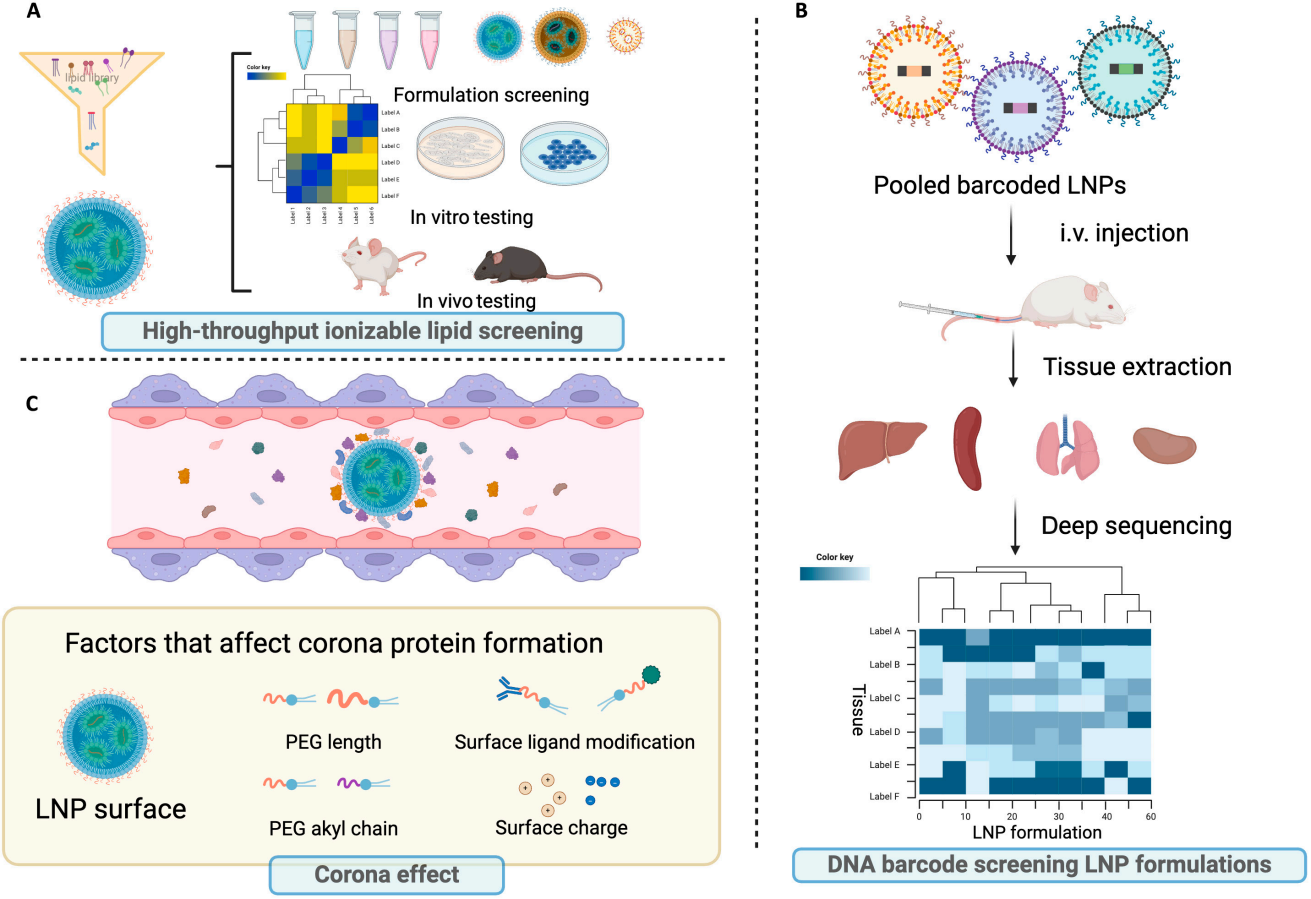
Schematic illustration of innovative strategies for accelerating LNP design. (A) Schematic illustration of high-throughput screening of ionizable lipids. (B) Barcode-based screening of LNP formulations for the advanced targeting efficiency. (C) Corona effect and the formation mechanisms for LNPs. Created with BioRender.com.

High-throughput screening method has been utilized for the design and optimization of ionizable lipid library since 2000s and reviewed in the “Ionizable cationic lipid” section (Fig. 10A). However, obtaining in vivo delivery efficacy data for over 1,000 lipids costs massive effort and poses a significant challenge due to the extensive workload and the complexity of animal profiles. To address this, Dahlman and colleagues [88] introduced a barcode system capable of effective formulation and material selection among over 100 LNP formulations using a single animal. As illustrated in Fig. 10B, each LNP formulation carries a unique DNA barcode sequence. Various formulated LNPs can be combined and intravenously injected into a single mouse. The percentage of different LNPs in different tissues can be calculated by deep sequencing the barcode percentages present in those tissues. Based on this approach, they characterized 250 LNPs, identifying two formulations for effectively delivering to endothelial cells [89]. One of these LNPs exhibited excellent performance in gene knockdown through delivering Cas9 mRNA and an sgRNA. Furthermore, Dahlman’s group developed a multi-omic nanoparticle delivery system that transcends tissue-level targeting to the cellular level. Since cells are composed of multiple transcriptional states, this system allows for the measurement of LNP-mediated RNA delivery at the cellular level. Dahlman and colleagues [90] employed single-cell nanoparticle targeting-sequencing (SENT-seq) to quantify dozens of LNP delivering barcode DNA and mRNA to different cell types. A startup company, Guide Therapeutics, was born out of this technology and later acquired by BEAM Therapeutics. Leveraging the barcode technique, Guide Therapeutics could search for effective LNPs to deliver therapies to any tissue and cell at a rate 15,000-fold higher than traditional experiments, proving its superior ability to accelerate the translation of mRNA therapeutics with a broader range. This barcode-based procedure has been widely adopted in LNP screening to generate in vivo efficacy data [43,46,91–93] expanding beyond DNA barcodes to include peptide barcodes [94]. Most recently, Xue et al. [95] utilized a barcoded DNA (b-DNA)-based high-throughput LNP screening system to investigate a combinatorial cationic degradable (CAD) lipid library, evaluating ionizable lipid chemical structure–activity relationships for pulmonary delivery. This approach facilitates the exploration of numerous nanoparticles in a single animal, leading to the identification of LNP-CAD9 as the lead candidate for pulmonary mRNA delivery, with approximately 90% of total luciferase expression observed in the lungs. As an ultimate applicable outcome of ionizable lipid and LNP formulation library high-throughput screening, they validated the efficacy of this platform by effectively co-delivering Cas9 mRNA/vascular endothelial growth factor receptor 2 (VEGFR2) sgRNA, which reduced VEGFR2 expression in lung endothelial cells. This demonstrates the therapeutic potential of antiangiogenic therapy for suppressing tumor growth and prolonging survival in a lung tumor model of female mice. Their findings further illustrate that high-throughput barcoding technology can serve as an efficient and effective screening tool for identifying structurally distinct nanoparticles for delivery to the lungs outside of the liver.

Interestingly, all of these utilizations of barcodes have laid the foundations for creatively and effectively searching for optimal drug delivery systems for mRNA therapeutics based on materials and formulations, and have been actively applied for lipid library screening for extrahepatic targeting until most recent time. However, even proved highly potent in early-stage screening of LNP, barcode-based approach may not guarantee optimal LNP selection for therapeutic mRNA delivery due to following facts: (a) the huge difference in the molecular size between therapeutic mRNA (usually around 2,000 to 5,000 nucleotides) and barcodes (usually around 60 to 100 nucleotides), resulting in diverse physicochemical properties of formulated LNPs, especially un-uniformity in encapsulation efficacy, which plays an essential role in determining the in vitro and in vivo performance of LNP delivery systems, and (b) the small size of the barcode also risks leakage from the LNPs during bench formulation or in the systemic circulation after administration, causing misleading results in tissue distribution and impairing the accuracy for barcode-based LNP selection. Therefore, developing a more stable and representative selection process for high-throughput LNP screening remains highly expected for more accurate acceleration of the translation for LNP-mRNA therapeutics.

Another aspect related to the in vivo performance of LNP-mRNA remains in the mist. When administrated into the body, nanoparticles absorb various proteins and generate a phenomenon known as the corona effect, as we briefly introduced in the “Phospholipid and cholesterol” section. Different LNPs form distinct protein coronas that target different tissues (Fig. 10C). For example, ApoE facilitates liver targeting, vitronectin enhances tumor cell-mediated LNP delivery, and fibrinogen augments LNP targeting to the lungs [96]. Consequently, corona adsorption is closely associated with nanocarrier coating and dynamics. In the case of LNPs, PEG length and alkyl chain affect both surface chemistry and PEG shedding rate [67], thereby influencing the surface coronation and targeting tissues. Pegylation plays a crucial role in determining the in vivo destination of LNPs and their payloads, suggesting its significance for LNP-mRNA therapeutic applications. Despite all the strides made, the underlying mechanisms and relationships between tissue targeting properties of LNP and protein corona formation remain elusive. Certain occurrences, such as the alteration of corona formation, have resulted in changes in tissue accumulation, yet a proper explanation is lacking [70]. Previous attempts illustrated that specific protein may serve as the nutrition or major supplement for the organs or the cells. There is also an explanation related to the receptor-mediated recognition, for which the most commonly acknowledged one is LDLR–corona protein–ApoE assisted liver targeting of LNP. However, various LDL receptors were identified other than ApoE, such as ApoA1. Basic knowledge related to physiology and pharmacodynamics remains to be explored. Therefore, while we appreciate the creativity of the concept of corona, it currently serves no more than a hypothesis in a long journey to promote LNP-mRNA into clinical stage. The lack of a liable detection technique also hinders the understanding of the protein corona phenomenon and stalls its development. Enabling in situ analysis of protein corona formation is crucial since current post-analysis via centrifugation separation and mass spectrometry is insufficient to unveil the authentic situation of protein adsorption around LNPs in the blood circulation. In a recent work, Zhan and colleagues [97] optimized the extraction of the corona in incubating serum by applying chromatography containing PEG-scFv (single-chain variable fragment). This facilitated the separation of pegylated nanoparticles and proteins while minimizing the depletion of protein corona components. Last but not least, predicting the in vivo performance of the corona protein formation around LNP, especially the stereochemistry and conformation of the absorbed proteins, which are essential to the cell-specific internalizations and successful translation of actively targeted LNP-mRNA, remains challenging.

In summary, the constant growth of lipid materials and nanoparticle drug delivery systems has inevitably prospered the evolution of mRNA therapeutics. The integration of various types of ligands, including small molecules, peptides, or antibodies, with nanoparticles, represents a powerful approach in drug delivery, enabling precise targeting and enhanced therapeutic outcomes. Small molecules are prioritized in economically friendly and readily applicable processes, while peptides and antibodies succeed in terms of complicated interactions with physiologically natural receptors, leading to higher and more precise targeting efficacy. Overall, the utilization of these materials, chemistry, and active targeting strategies accelerates the advancement of LNP-mRNA therapeutics in treating diseases and facilitating genetic regulation, revolutionizing the field of drug delivery and opening new avenues for the treatment of a wide range of diseases.

## Manufacture and Safety Concerns for LNP-mRNA Therapeutics

### Manufacture and storage

A major unmet need in advancing LNP-mRNA therapeutics is the development of technology to produce precisely defined formulation scaling from discovery to commercial manufacturing while meeting the stringent manufacturing standards of the pharmaceutical industry (good manufacturing practice, GMP).

The identification, optimization, and manufacturing requirements for mRNA molecules in mRNA-LNP medicines differ significantly across various therapeutic types. For the prevention and treatment of infective disease, common mRNA vaccines encode and express immunogenic antigen of interest. A notable benefit of mRNA technology lies in its capacity to easily design and modify antigens by introducing changes in nucleic acids. This process is comparatively straightforward when compared to the bioengineering challenges associated with distinct proteins or peptides. For example, the sequence identification and optimization of COVID-19 mRNA vaccines have been intensively explored [98]. Additionally, the mRNA-1653 vaccine exemplifies the capability of a single vaccine to simultaneously target two distinct pathogens. This vaccine combines two mRNA species, specifically directed toward the F protein of human metapneumovirus (hMPV) and parainfluenza virus type 3 (PIV3), proving the flexibility of mRNA vaccines [99]. For cancer immunotherapy, especially personalized cancer vaccines, patient-specific mRNA neoantigens are identified by next-generation sequencing, which could be time and cost consuming. In gene-editing therapies, mRNA typically encodes genome-regulating tools like Cas9 or dCas9 proteins, incorporating multiple components with distinct functions. In contrast to vaccines, gene-editing tool mRNA molecules typically possess an ultra-length of at least 5 kDa, presenting challenges in terms of manufacturing and purification processes. Moreover, gene-editing regulation therapy demands a dosage of 10 to 100 mg of mRNA per dose, which is significantly higher by a factor of 1,000 when compared to mRNA vaccines. This stark difference necessitates unique considerations and substantial efforts to ensure a seamless large-scale production process. In summary, it is crucial to tailor considerations accordingly for each therapy during the identification, optimization, and manufacturing stages of mRNA.

Successful clinical translation of LNP-mRNA therapeutics not only hinges on mRNA but also is equally reliant on the critical aspects of formulation discovery and the scaled manufacturing process LNPs. Starting from the discovery stage, LNP physicochemical parameters are optimized on a smaller scale for the enhancement of RNA delivery efficacy by tuning the lipid chemical structure, modifications of mRNA, and lipid ratio and lipid/mRNA ratio adjustment [43,62,100,101]. Once a fixed LNP formulation is identified, the requirement to implement commercial-scale production with acceptable cost and rate becomes different and harsher. Scaling up the synthesis of LNPs to GMP level can present challenges, including the high cost of raw materials, overly complex designs, and risks of endotoxin contamination, among others [102]. Efforts have been input in investigating the stable and sustainable process for LNP-mRNA manufacturing. Taking lessons from the development of Dox-liposome, Liu and Meng [103] suggested that overcoming the challenges of multiparameter LNP production could be achieved by leveraging the potential of artificial intelligence (AI) and machine learning to determine the optimal engineering parameters. Additionally, employing orthogonal design of experiments (DOE) may offer an alternative for complex CMC development of LNP-mRNA medicines. With precise control and optimization of parameters such as channel design and flow rate, microfluidics could offer feasible mixing solutions for both laboratory and industrial development, providing practical advantages and potentials over traditional methods. Mitchell’s group from Pennsylvania University designed a microfluidic chip system to enable LNP-mRNA production at both small discovery and large clinical testing scales (17L/h). Their innovative silicon scalable lipid nanoparticle generation (SCALAR) chips were suitable for high-temperature sterilizing methods. The efficacy of LNPs generated using the SCALAR chips for mRNA transport in vivo was comparable to that of LNPs generated using the regular and currently applied polydimethylsiloxane (PDMS) chip. This addressed the gap between formulation techniques at small-scale discovery stage and GMP-level production [104].

We have extensively summarized preclinical explorations utilizing antibody-conjugated LNPs to enable mRNA therapeutics for various applications in the “Antibody modifications for ligand–receptor-mediated tissue targeting” section. In particular, CMC plays even more crucial roles in successful development and translation for complex formulations such as antibody–LNPs [105]. It is well acknowledged that for nanomedicines like anti-LNPs, minor changes in manufacturing conditions can result in substantial changes in the final product’s properties, making it difficult to maintain the products’ quality, safety, and efficacy and control the process conditions and production scales. For instance, slight adjustments in the process conditions can impact the size, shape, surface charge, and other vital quality attributes of nanoparticles. These factors, in turn, influence their biodistribution, clearance, and therapeutic effectiveness [106]. Likewise, scaling up the production process from research to an industrial scale can introduce variability that requires careful control to maintain consistent product quality. Thus, a thorough comprehension of the manufacturing process and the implementation of robust control strategies are essential for the successful development and commercialization of antibody–LNP–mRNA drug products [107].

Alongside with the scaling process and manufacturing, characterizing the quality of LNP production is another pivotal factor in the development of mRNA therapeutics, since the successful employment of the scaling-up relies on appropriate analytical methods and quality standards for process control and product characterization. On the other hand, the quality evaluation entails risk assessment of residual raw materials, establishment of testing and control strategies, and analysis of potential residues of elemental impurities following the guidelines outlined in ICH Q3D [108]. Current impurities associated with LNP-mRNA products have been identified, including double-stranded RNA, truncated mRNA, uncapped mRNA, and double-stranded DNA template for mRNA modality, along with free lipid components and lipid-related degraded or oxidized chemicals with LNP systems [109].

Besides progress and on-shelf quality control in GMP processes, challenges persist in LNP storage and transportation. Due to the unstable nature of mRNA and easy corruption of lipid-based delivery systems, commonly applied strategy relies on −80 °C or −20 °C cold chain, just as Moderna and Pfizer-BNT COVID-19 mRNA vaccines. However, the cold-chain approach limits broader therapeutic applications, and the harsh storage temperature and undesirable cryoprotectants also impede the quality of LNP-mRNA upon thawing. Lyophilization and cryoprotection of LNP-mRNA have been the most attractive topic for empowering wider applications. Kim et al. [110] discovered that in a PBS buffer with 10% (w/v) sucrose added as a cryoprotectant, LNP-mRNA could be stored stably at −20 °C for at least 30 days. Therefore, lyophilization is a major option for the LNP-mRNA therapeutics and substantial in vivo bioavailability could be retained. To date, research and development on the transport stability and optimization conditions of LNP-mRNA medicines remain limited. Customized services may be necessary for different types of mRNA and LNPs. The challenges associated with transporting and storing such downstream processes primarily rely on the efforts of contract development and manufacturing organization (CDMO) companies within the pharmaceutical supply chain. More efforts should be directed toward downstream processes, including CMC, quality control, transportation, and storage, to expedite the translation and widespread applications of LNP-mRNA therapeutics.

### Characterization techniques

**Fig. 11.**
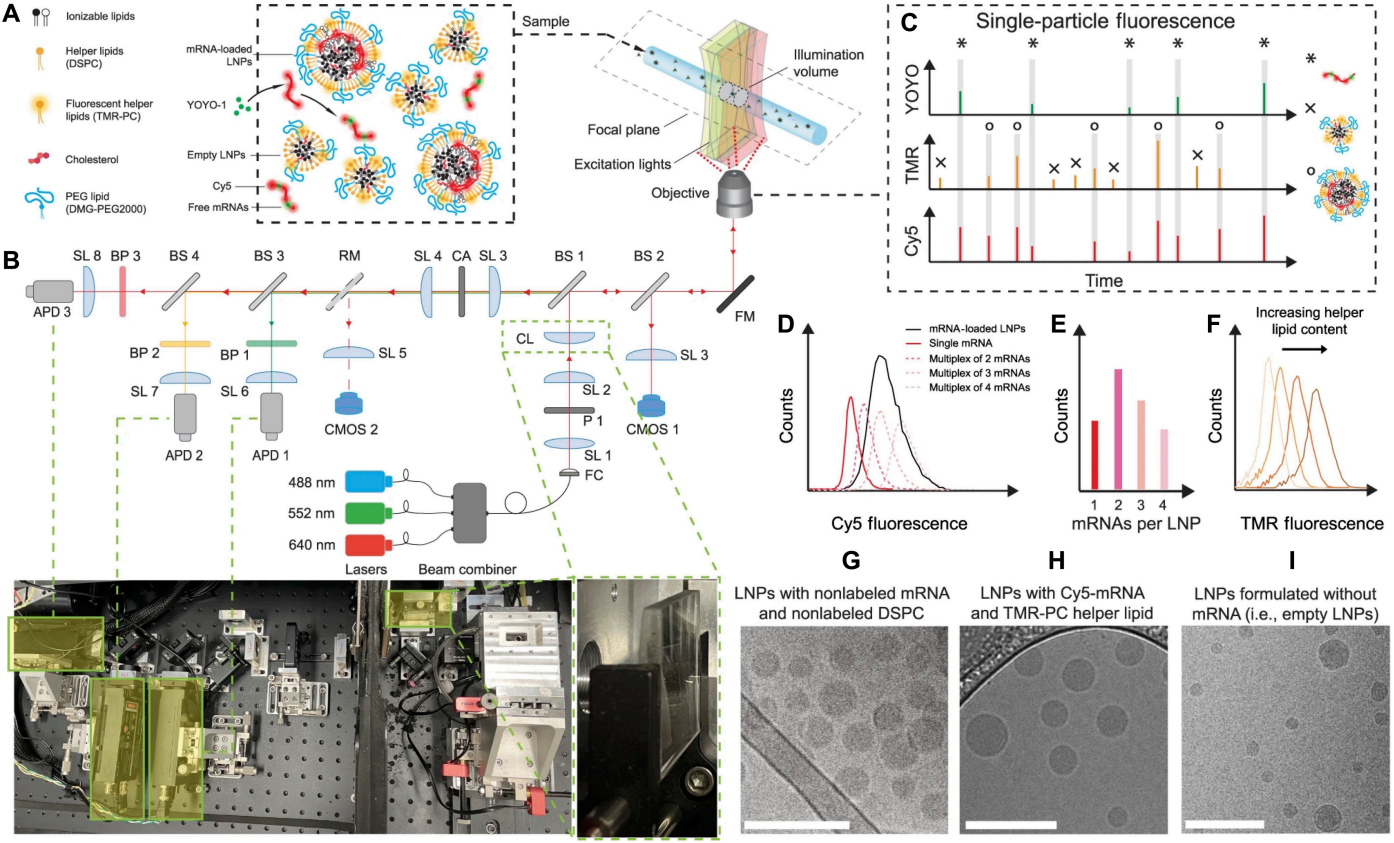
Instrumentation of multi-color CICS platform and methodology for characterization of LNP formulations. Reproduced with permission [111,133]. Copyright 2022, Springer Nature. (A) Three fluorescent tags were used for single-particle fluorescence detection and categorization: Cy5 tag for mRNA, TMR tag for help lipids, and YOYO-1 for unloaded mRNA. (B) Instrumental setup of three-color CICS. (C) For all the detecting species of interest, the fluorescence was classified as mRNA-loaded LNPs (circles, TMR-Cy5 coincident), empty LNPs (crosses, TMR only), and free mRNAs (asterisks, Cy5-YOYO-1 coincident). (D and E) The Cy5 intensity profile of single free mRNA molecules, and the profile shifted right when multiplexed mRNAs were expected in LNPs. (F) TMR intensity profiles of LNP formulations correlate with their relative helper lipid content. (G to I) Cryo-TEM images of mRNA LNPs of the benchmark formulation at pH 7.4. (G) LNPs formulated with nonlabeled mRNA and nonlabeled DSPC. (H) LNP formulated with Cy5-mRNA and 0.5% (mol % to total lipid content) TMR-PC. (I) Empty LNPs formulated in the absence of mRNA. All scale bars, 200 nm.

Insufficient systematic analysis and tracking of LNP-mRNA both on the shelf and in vivo impede the broader applications for mRNA medicines. Conventional characterizations primarily rely on size, zeta potential analyzed by dynamic light scattering, and encapsulation efficiency assessed through fluorescence methods. However, these methods often fall short of meeting the stringent standards required for pharmaceutical production. Research on the proportion of empty particles is an ongoing endeavor, yielding significant findings in academic settings. However, the translation of these discoveries into industrial production has been limited. Li et al. reported a method based on the multi-laser cylindrical illumination confocal spectroscopy (CICS) technique to examine mRNA and lipid contents in LNP formulations at the single-nanoparticle level [111,133]. The mechanism and methodology of their equipment and technique are shown in Fig. 11. They differentiated unencapsulated mRNAs, empty LNPs, and mRNA-loaded LNPs by employing coincidence and quantitative analysis of fluorescent tags on various LNP components and fluorescence of single mRNA molecules. Their findings indicated that a frequently cited benchmark formulation, incorporating ionizable lipid DLin-MC3, predominantly accommodates two mRNAs per loaded LNP. The presence of empty LNPs ranged from 40% to 80%, contingent on the specific assembly conditions. By altering the molar ratio of pegylated lipid and N/P ratio [this ratio refers to the ratio between positively charged amine groups (N) of the LNP and negatively charged phosphate groups (P) of RNA], they demonstrated a mechanism of kinetically controlled assembly that governs the distribution of payload and capacity within LNP. The discovery serves as a comprehensive understanding of the molecular assembly of LNP-mRNA. However, it inevitably relied on fluorescence labeling (Cy5 or YOYO) of the nucleic acid to distinguish between encapsulated and free mRNA, hindering the applicability of in situ analysis on the scalable production of mRNA-LNP. At the same time, Wyatt Technology provides an alternative for quantified payload analysis for LNP-RNA based on size separation system, size exclusion chromatography with multi-angle light scattering (SEC-MALS), or more often field-flow fractionation with multi-angle light scattering (FFF-MALS), measuring high-resolution size distributions, particle concentration, and morphology in a single run. However, high cost associated with the analysis hinders its widespread adoption. Systematic analysis and regulatory release criteria for LNP-mRNA remain incomplete. Addressing these challenges is crucial for advancing mRNA therapeutics to clinical investigation and approval.

### In vivo performance and safety concern

Besides manufacturing, storage, and carriage, the in vivo performance, also known as adsorption, distribution, metabolism, and elimination (ADME), of LNP-mRNA modality remains unclear and under evaluation. For vaccines like the approved severe acute respiratory syndrome coronavirus 2 (SARS-CoV-2) vaccines, understanding the ADME of LNPs and their respective lipids in specific tissues such as muscles, liver, spleen, and urine is crucial. Moreover, when LNP-mRNA modality was incorporated as genetic regulation therapeutics, much higher doses (an almost 1,000-fold higher over vaccine) were required. Furthermore, approaches such as protein replacement therapies recommend repeated administration to realize therapeutic effects, which probably leads to a substantial burden on healthcare systems and poor patient compliance. Thus, ADME analysis is more critical for the LNP-mRNA therapeutics, in terms of safety, tolerance, and minimizing off-target genetic editing. Nevertheless, there has been insufficient explorations in this area.

For instance, while Moderna conducted several analysis on the degradation products and predicted several metabolites and the circulating profile in vivo for their ionizable lipid SM-102, which has been utilized in COVID-19 vaccines [111], these studies are limited, with only one-step degraded product analyzed in the serum. Similarly, in the clinical profile of the first FDA-approved in vivo LNP-mRNA-mediated gene-editing therapy for the treatment of transthyretin amyloidosis, only the ionizable lipid LP01 and pegylated lipid DMG-PEG2k were assessed in the plasma and urine, with no systemic analysis of the biodistribution of the degradation products of the ionizable lipid or mRNA in other major organ such as the spleen or lung [27]. For future mRNA-based therapies, thorough ADME evaluations targeting novel ionizable lipid and LNP formulations are highly demanded, addressing the relevant research and validation in accordance with the standards of novel drug ADME testing.

Another challenge that impedes the extensively successful translation of LNP-mRNA therapeutics is species-dependent in vivo delivery facilitated by LNPs. In a recent work, Dahlman and colleagues [112] utilized the barcoding system refered as species agnostic nanoparticle delivery screening (SANDS) method to quantify how 89 distinct LNPs facilitated liver accumulations of mRNAs, in humanized and privatized murine models, hoping to validate if the LNP delivery efficacy in mice could successfully translate to NHPs and clinical trials. However, the physiological structure of mice evidently differs from the primates, implying intensive and direct evaluations on the NHPs and human. This detail is crucial yet lacks substantial research investment and attention. Recently, Cullius’ group illustrated that optimizing the surface and physicochemical parameters of LNPs, with reduced particle size to around 50 to 60 nm and the increased PEG concentration to around 2.2 to 2.8%, proved to be more effective for NHP rather than the mouse [113]. Research efforts must address the translation of LNPs’ delivery efficacy from murine models to NHPs and clinical trials.

Additionally, for local injection, such as intravitreal applications in ocular therapy, rather limited effort and clinical progress have been made since these years. Blindness is detrimental to the human health, while posterior ocular diseases such as glaucoma or age-related macular degeneration (AMD) account for over 80% of partial or total blindness. Genetic regulation for treating ocular diseases has been under investigation since the 2000s. While multiple virus-based delivery systems for genetic material-based drugs have undergone clinical trials, LNP-mRNA therapeutics for ocular diseases remain in the preclinical stage. The anatomical differences among the eyes of various species, such as mice, rats, rabbits, NHPs, and human, result in hurdles in the translation of the optimal formulation of LNPs with desired efficacy from smaller animals to clinical applications. Besides efficacy, factors such as tolerance and immune activation also vary across species and must be taken into consideration. All in all, while issues such as CMC, storage, pharmacology, pharmacokinetics, and pharmacodynamics among species have been addressed to some extent, broader implementation of LNP-mRNA therapy still unyieldingly demands for further research and development efforts.

## mRNA as Therapeutics: A Clinical Perspective

Two LNP-mRNA vaccines have successfully piloted the fight against the SARS-CoV-2 pandemic and taken the lead in achieving international regulatory approval. Ideally, mRNA as a drug modality could facilitate protein production systemically or locally. In this case, a broad range of disease indications and categories of proteins could be selected and investigated including enzymatic proteins, receptors, secreted proteins like anti-VEGF, and gene-editing proteins like Cas9. Apart from the application of naked VEGF-mRNA therapeutics for heart failure treatment from AstraZeneca (AZD8601, in partnership with Moderna), most protein replacement therapy involving LNP-mRNA medicines has primarily undergone intensive preclinical exploration. It is noteworthy that despite substantial research efforts, only a select few cases have advanced to the stage of clinical translation. Significantly, half of these trials were terminated upon the conclusion of phase I. Ongoing clinical trials via LNP-mRNA to facilitate protein replacement therapies include mRNA-3927 from Moderna to facilitate enzyme therapy to treat propionic acidemia (NCT04159103), MRT5005 from Translate Bio for treating cystic fibrosis and restoring lung function (NCT03375047), and RCT1100 from Recode Therapeutics to treat primary ciliary dyskinesia (PCD) caused by pathogenic mutations in the DNAI1 gene (NCT05737485). One highlighted preclinical investigation related to protein replacement therapy and immunotherapy is from Weissman’s group partnered with Acuitas Therapeutics to utilize humanized interlukin-10 (a neuroprotective factor) mRNA-loaded LNP (hIL-10 mRNA-LNP) via intraspinal cord administration to induce neuroprotection and functional recovering in the rat spinal cord contusion injury model [114]. The fluorescent imaging results demonstrated that LNP-encapsulated enhanced green fluorescent protein (eGFP) mRNA could facilitate potent transient protein expression up to 21 days after intraspinal cord administration in both intact and injured rat spinal cord. Upon intralesional administration of hIL-10 mRNA-LNP, a noteworthy decrease in microglia/macrophage activity within the injured spinal segment was observed. This was accompanied by the down-regulation of pro-inflammatory cytokines such as tumor necrosis factor-α (TNF-α) and CCL3 and an up-regulation of the central nervous system (CNS) anti-inflammatory cytokine IL-6. This highlighted preclinical research finding suggests that the intervention involving protein replacement, coupled with immunotherapy and inflammatory regulation using LNP-mRNA medicines, holds promise for the restoration of neuron function and injury repair in localized tissue areas, such as in the spinal cord or in the posterior ocular segment. For example, this approach could have clinical applications in conditions like glaucoma, where the neuroprotection of retinal ganglion cells via LNP-mRNA could offer therapeutic benefits to decelerate the glaucoma progression. Both research and industrial endeavors are required to advance LNP-mRNA protein replacement therapy for clinical translation in the near future.

Additionally, this section briefly highlighted other three categories of mRNA applications, which are already in clinical trials (briefly summarized in Table) for treating infectious diseases and facilitating cancer immunotherapy and genetic therapy.

**Table.** Clinical trials of LNP-mRNA vaccines and therapeutics

Sponsoring institution	Drug name	Disease indication	Therapeutic approach and vaccine targets	Trial number	Status
Moderna	mRNA-1893	Zika virus	prME structural protein	NCT04064905 NCT04917861	Phase II ongoing
Moderna	mRNA-1325	Zika virus	Anti-Zika monoclonal antibody	NCT03014089	Phase I completed
Moderna	mRNA-1440 (VAL-506440)	Influenza	Hemagglutinin from H10N8 strain	NCT03076385	Phase I completed
Moderna	mRNA-1851 (VAL-339851)	Influenza	Hemagglutinin from H7N9 strain	NCT03345043	Phase I completed
Sanofi Pasteur	VAV00019	Influenza	Hemagglutinin from H3 strain	NCT05829356	Phase I ongoing
GSK	GSK3903133A	Rabies	Rabies glycoprotein G	NCT04062669	Phase I completed
CureVac	CV7202	Rabies	RABV-G protein	NCT03713086	Phase I completed
Moderna	mRNA-1345	RSV	RSV prefusion F protein	NCT04528719	Phase I ongoing
Moderna	mRNA-1388 (VAL-181388)	Chikungunya virus	Antigenic proteins	NCT03325075	Phase I completed
Moderna	mRNA-1647	CMV	CMV glycoprotein H	NCT03382405	Phase I completed
AstraZeneca Moderna	AZD8601	Heart failure, male subjects with type II diabetes, patients with coronary artery bypass surgery	VEGF-A	NCT02935712 NCT03370887	Phase II completed
Moderna	mRNA-3927	Propionic acidemia	Alpha and beta subunits of propionyl-CoA carboxylase	NCT04159103	Phase I ongoing
Translate Bio	MRT5005	Cystic fibrosis	Human CFTR	NCT03375047	Phase I/II ongoing
ReCode Therapeutics	RCT1100	Primary ciliary dyskinesia	DNAI1	NCT05737485	Phase I ongoing
MedImmune LLC, AstraZeneca	MEDI1191	Advanced solid tumors	IL-12	NCT02888756	Phase I completed
Moderna	mRNA-2752	Relapsed/refractory solid tumor malignancies or lymphoma	OX40L, IL-23, and IL-36γ	NCT03739931	Phase I ongoing
BioNTech	BNT-122	Untreated advanced melanoma	Neoantigen	NCT03815058	Phase II ongoing
BioNTech	BNT-122	NSCLC	Neoantigen	NCT04267237	Phase II/III ongoing
BioNTech	BNT-122	PDAC	Neoantigen	NCT05968326	Phase I completed, phase II recruiting
BioNTech	BNT-114	TNBC	Neoantigen	NCT02316457	Phase I completed
Intellia Therapeutics	NTLA-2001	hATTRv-PN, hATTRv-CM, hATTRwt-CM,	SpCas9	NCT04601051	Phase I ongoing, phase III approved
Intellia Therapeutics	NTLA-2002	HAE	SpCas9	NCT05120830	Phase I recruiting
Verve Therapeutics	VERVE-101	HeFH, ASCVD, uncontrolled hypercholesterolemia	Adenine base editor	NCT05398029	Phase I recruiting

RABV, rabies virus; RSV, respiratory syncytial virus; CMV, cytomegalovirus; VEGF, vascular endothelial growth factor; CFTR, cystic fibrosis transmembrane conductance regulator; IL-12, interleukin-12; IL-23, interleukin-23; IL-36γ, interleukin-36γ; PDAC, pancreatic ductal adenocarcinoma; TNBC, triple-negative breast cancer; NY-ESO-1, New York-ESO 1; MAGE-A3, melanoma-associated antigen A3; TPTE, trans-membrane phosphatase with tensin homology; hATTRv-PN, hereditary transthyretin amyloidosis with polyneuropathy; hATTRv-CM, hereditary transthyretin amyloidosis with cardiomyopathy; hATTRwt-CM, hereditary transthyretin amyloidosis with wild-type cardiomyopathy; HAE, hereditary angioedema; HeFH, heterozygous familial hypercholesterolemia; ASCVD, atherosclerotic cardiovascular disease.

### Infective diseases

mRNA vaccines against infective diseases have been investigated intensively around even long before the COVID-19 pandemic. Here, we will not review broadly regarding SARS-Cov-2 vaccines; only the most worth-mentioning mRNA vaccines are detailed since COVID-19 LNP-mRNA vaccines have been comprehensively discussed elsewhere. A number of clinical studies regarding mRNA vaccines to combat highly contagious diseases including influenza, ZIKA, rabies, and even HIV are being conducted. One of the most standout mRNA companies, Moderna, has set up pipelines against most of these fatal viruses, of which over 20 have been approved to translate in clinical trials. Compared to the placebo-vaccinated group, their mRNA-1325 (NCT03014089, phase I completed) showed good tolerability in healthy adults with poor Zika virus-specific neutralizing antibody responses. Most recent advances updated that their second mRNA vaccine, mRNA-1893 (NCT04064905, NCT04917861, phase II), generated comparable neutralizing antibody titers to mRNA-1325 (NCT03014089, phase I) at 1/20th of the dose. It also proved extensive protection for ZIKA-challenged NHPs [115,116]. In late 2021, mRNA-1893 also finished phase I trial and has expressed great tolerability and now it is being examined in phase II trials.

Influenza also attracted scientists’ and research investors’ attention due to its high prevalence. mRNA-1440 (previously VAL-506440) and mRNA-1851 (VAL-339851) from Moderna were LNP-mRNA vaccines expressing full-length hemagglutinin from H10N8 and H7N9 strain A variant, respectively, and have shown promising results in their separate phase I trials (NCT03076385 and NCT03345043). Both vaccines elicited significant seroconversion and excellent seroprotection with limited adverse effects, such as pain at the injection site and common cold-like symptoms, indicating acceptable safety and tolerability [117,118]. Other than Moderna, multinational corporation pharma companies like Sanofi have also made substantial efforts in mRNA flu vaccine, conducting phase I trial against the H3 variant (NCT05829356). GSK has spotted mRNA as a vaccination strategy and explored a self-amplifying mRNA-loaded LNPs for rabies infection, completing a phase I trial (NCT04062669) using LNP-mRNA system. CureVac has made significant progress in the development of a rabies mRNA vaccine (CV7201), with mRNA encoding the glycoprotein of the rabies viruses. In their phase I study, over 70% of the participants elicited antigen-specified antibody response (NCT02241135) [119,120]. With optimized LNP encapsulating the same mRNA antigen, CureVac has iterated rabies vaccines and finished the phase I trial with good tolerability (NCT03713086). Other infective diseases targeted with mRNA vaccine interventions in clinical trials include respiratory syncytial virus (NCT04528719), Chikungunya (NCT03325075), and cytomegalovirus (NCT03382405), most of which stay at or just completed phase I stage.

In summary, these clinical studies suggest that LNP-mRNA vaccines are being well developed and will continue as a promising strategy against infectious diseases. However, future efforts are still expected in the advanced antigen and immunostimulatory adjuvant against the difficult-to-deal-with diseases. For instance, the route paving toward prevention and protection against AIDS never stops. Unfortunately, several studies of AIDS mRNA vaccines that went into clinical trials (NCT02888756 and NCT00672191) failed to demonstrate evident antiviral efficacy and effective control of acute HIV infection. Thus, cross-disciplinary strategies are still needed in developing potent antigen sequences and adjuvants for the improved vaccinations.

### Personalized cancer immunotherapy

mRNA technologies have emerged as promising tools for cancer immunotherapy, of which some succeed in clinical translation. Several cases have conducted clinical trials, and the majority are about to complete phase I, including treatment against solid malignant tumors (MEDI1191, NCT03946800) from MedImmune and AstraZeneca and solid malignant tumors/lymphoma (mRNA-2752, NCT03739931) from Moderna. The most advanced strategy is personalized vaccines for cancer intervention [84]. These vaccines are generated through a protocol as follows: (a) acquire the patient’s healthy and tumor tissues, (b) get the tissues precisely sequenced and bioinformatically analyzed for individual tumor-specific neoepitopes, and (c) produce mRNA vaccines encoding these neoepitopes [121]. One of the most uprising biotech companies, BioNTech, besides rapidly and successfully overcoming COVID-19 pandemic with collaboration, acts prominently in the development of a personalized cancer vaccine platform. BNT-122, also known as Autogene cevumeran, has completed phase I studies against melanoma (NCT03815058) and non-small cell lung cancer (NSCLC; NCT04267237). Recently, a groundbreaking study reported that this personalized mRNA cancer vaccine has been applied to treat a highly malignant form of pancreatic cancer, known as pancreatic ductal adenocarcinoma (PDAC). This mRNA cancer vaccine, encoding 20 neoantigens, was encapsulated and delivered by LNP via intravenous injection. The outcome showed that in 16 PDAC patients after surgical resection, in combination with chemotherapy (mFOLFIRINOX regimen) and immune checkpoint therapy (anti-PD-L1 monoclonal antibody), a significant T cell response was observed in 50% of the patients, suggesting that this personalized mRNA vaccine could trigger substantially enhanced immune responses and opened a new avenue for this detrimental disease [122]. Besides BNT122, some vaccines originated from BioNTech have promising therapeutic effects in preclinical cancer models [123,124] and are currently in the early stages of clinical evaluation for breast cancer (NCT02316457) and melanoma (NCT04526899, phase II).

Despite promising findings, hurdles remain for the widespread adoption of mRNA-personalized vaccines in oncologic therapy. These include the complexity of identifying suitable antigens, the potential for tumor immune escape, as well as the requirement for scalable trials to confirm durable safety and efficacy. Additionally, current immunogenicity of LNP may not be robust enough for antitumor effects. For example, dosage for adult single shot of COVID-19 mRNA vaccine is approximately 20 μg, despite its high antibody levels and efficacy rate. However, there are still limitations in its cross-species protective rate and duration (around 6 months). This also indicates that its cellular immune responses still require enhancement, especially for cancer immunotherapy. Hence, the development of a more robust adjuvant is needed. On the one hand, it would activate a stronger CD8 T cell response to directly kill tumor cells. On the other hand, it could also stimulate natural immunity, provoking natural killer (NK) cells and neutrophil engagement to improve the tumor microenvironment and overall antitumor effect. Potent CD8 T cell activations, neutrophils, and NK engagement, as well as the immunogenic regulations of the tumor microenvironment, are also expected.

### Gene editing

Recently, gene-editing therapy represents another rapidly growing area of mRNA-based drugs, empowered by the discovery of the CRISPR-Cas9 system. The discovery of the CRISPR-Cas9 system, in which the mechanism was adapted from bacterial defense and now has been adapted to generate double-stranded genomic DNA breaks in eukaryotic cells for in vivo gene regulation [125], empowers precise gene regulation and permanent cure for genetic disorders [126]. Two components are involved proactively in gene-editing: nuclease responsible for DNA cleavage and guide RNA directing the nuclease, usually Cas or dCas, to precisely locate the cutting site [127,128]. When applied in vivo, both components need to be delivered into the targeting organ and cells, in which the nuclease was mostly directly sent into the cells or encoded via a DNA plasmid. Pure Cas nuclease facilitates less satisfied editing efficacy, whereas plasmid-mediated genome editing potentially leads to off-target genomic DNA cut as a result of undesired durable expression of the Cas protein [129]. As such, mRNA encoding Cas protein becomes an attractive alternative, as protein expression exists transiently. Aside from CRISPR-Cas-based genome editing, improved editing tools including base and prime editors, which facilitate precise nucleotide correction, have elucidated enhanced target specificity and therapeutic efficacy in preclinical trials. Both platform technologies of gene-editing molecular biology and LNP delivery systems assisting large mRNA payloads enable clinical translation of mRNA-based gene-editing treatments.

As for the delivery vehicles, LNPs loaded with mRNA have primarily been utilized to prompt transient protein expression, making them suitable for applications such as vaccines. However, for gene editing, a kind of protein replacement therapies, durable efficacy is essential, forming higher expectations for the LNPs that immense potent delivery and expression efficacy of the target mRNA. In 2023, the first CRISPR-based ex vivo gene therapy, exagamglogene autotemcel (exa-cel), got the FDA’s acceptance of Biologics License Applications (BLA) for treating sickle cell disease (SCD) and β-thalassemia. This landmark approval has stricken the whole field [130]. Currently, several other ex vivo and in vivo gene-editing therapies to treat hereditary transthyretin amyloidosis (hATTR), metabolic disorders, or retinal dystrophies are being clinically examined (NCT05398029, NCT05120830, NCT05885464, NCT03041324, NCT02702115, NCT04601051, and NCT03872479). Although most meteoric triumph has stepped out in ex vivo gene therapy, in vivo LNP-mRNA-assisted gene-editing therapeutics need extra effort. Only one representative case has completed the phase II clinical trial, NTLA-2001 from Intellia Inc., utilizing CRISPR-Cas therapy to treat ATTR [27]. In their phase I study (NCT04601051), they documented that a single intravenous administration of LNPs encapsulating a combined cargo of Cas9 mRNA and guide RNAs resulted in cleavage of the target mutant protein gene and an over 90% reduction in circulating mutant protein levels, and the down-regulation lasted until 28 days after administration without any adverse side effects. They recently completed the FDA clearance of the NTLA-2001 application, allowing them to initiate a pivotal phase III trial in the United States, marking the first in vivo CRISPR-based candidate to begin late-stage clinical development. As introduced above, because the mRNA is only transiently expressed, this approach succeeded in traditional and popular adeno-associated virus (AAV)-mediated delivery of Cas9 in terms of safety, where there may exist persistent expression of the nuclease long after the desired editing event. This first-ever clinical study demonstrated that CRISPR mRNA therapeutics rescue liver genetic disorders in humans. Following that, Verve Therapeutics achieved amazing therapeutic effects in the preclinical stage by applying LNP loading mRNA encoding base-editing tool to treat heterozygous familial hypercholesterolemia (HeFH) and cardiovascular disease [131,132], and their phase I clinical trial is actively recruiting (NCT05398029).

In spite of all these promising and remarkable milestones achieved for using LNP-mRNA as gene-editing therapeutics, challenges remain. Most gene therapy still needs AAV or lentivirus as delivery vehicles. Only countable nonviral systems, namely, LNPs, have successfully assisted mRNA gene-editing modality translated into the clinical trials, especially when coming to in vivo application. One of the largest limitations of the nonviral vehicles in vivo is instinct hepatic accumulations. As reviewed in the last section, although intensive research in extrahepatic LNP targeting has been investigated through the years with satisfied spleen or lung or localized organ targeting like ocular drug targeting, none of the cases moved forward clinically. Therefore, much more attention and effort may need to be drawn toward this direction. Platform technology such as extrahepatic drug delivery may largely empower mRNA-based genetic therapy to benefit the precise treatment of more and more diseases other than liver disorders and cardiovascular diseases.

## Conclusion and Future Scope

In the past three decades, academic and clinical advances coupled with the successful development of mRNA COVID-19 vaccines have illuminated the potential of mRNA therapeutics in disease treatment. This review aims to empower the development of mRNA therapy and medicines via enhanced LNP delivery systems. We introduced the state-of-the-art progress of LNP-mRNA therapeutic applications, coupled with the structure and design guidelines for LNP delivery systems, as well as the summary of preclinical and clinical trends for LNP-mRNA therapy. In particular, we provided a comprehensive and critical examination of the challenges and unmet requirements crucial for the successful translation and broad application of LNP-mRNA medicines.

It is worthy to mention that the primary obstacle encountered in transitioning from siRNA to mRNA delivery lies in unsatisfied RNA release, particularly with long-chain mRNA. This issue also renders non-LNP delivery systems undesirable for mRNA delivery, for its more rigid structure, resulting in insufficient release and reduced mRNA translation efficacy. In the meantime, it has also been mentioned above that several crucial goals must be achieved before the therapeutic potential of mRNA LNP is fully unlocked. Understanding the biological pathways and metabolism of both mRNA modality and lipid delivery systems not only benefits the efficacy of mRNA drugs but also addresses concerns over potential toxicity and unwanted immune response.

In addition, innovations in LNP delivery systems to target organs and tissues beyond the liver are imperative for expanding the scope of LNP-mRNA therapeutics to treat common and rare diseases. Ultimately, the quick and efficient implementation of LNP-mRNA therapy largely relies on the manufacture, storage, and quality control of the drug. Therefore, the development of modular, scalable GMP-level production facilities, coupled with systematic analysis of LNP-mRNA drugs, is essential to facilitate their global applications. Additionally, the deployment of thermostable LNP-mRNA formulations and lyophilization techniques would largely obviate the logistics and distribution challenges that could hinder the progress of LNP-mRNA.

With unprecedented mRNA COVID-19 vaccines, the unlimited potentials of LNP-mRNA have already been demonstrated. Continued innovations in mRNA biology, lipid chemistry, and LNP formulations, along with enhanced targeting drug delivery systems, and GMP scalation with systematic characterization on shelf and in vivo, coupled with long-term storage capabilities, could enable innovative LNP-mRNA-based therapies, offering new hope for many patients with unmet clinical needs. Future LNP-mRNA nanomedicines will necessitate increased precision, extended duration, and tolerable safety profiles to allow for chronic and multiple dosing, thus empowering this modality wider applications in the treatment of both common and rare diseases. This includes the strategies beyond vaccines, including cancer immunotherapies, protein replacement therapies, and genetic regulation-based therapies.
